# Interaction with Human Serum Proteins Reveals Biocompatibility of Phosphocholine-Functionalized SPIONs and Formation of Albumin-Decorated Nanoparticles

**DOI:** 10.1021/acs.langmuir.0c01083

**Published:** 2020-06-23

**Authors:** Irene Russo Krauss, Alessandra Picariello, Giuseppe Vitiello, Augusta De Santis, Alexandros Koutsioubas, Judith E. Houston, Giovanna Fragneto, Luigi Paduano

**Affiliations:** ^†^Department of Chemical Sciences and ^§^Department of Chemical, Materials and Production Engineering, University of Naples Federico II, Naples, Italy; ‡ CSGI, Center for Colloid and Surface Science, Sesto Fiorentino (FI), Italy; ∥ Jülich Centre for Neutron Science (JCNS) at Heinz Maier-Leibnitz Zentrum (MLZ), Forschungszentrum Jülich GmbH, Lichtenbergstrasse 1, 85747 Garching, Germany; ⊥ European Spallation Source ERIC, Box 176, SE-22 100 Lund, Sweden; # Institut Laue-Langevin (ILL), 71 avenue des Martyrs, BP 156, 38042 Grenoble, France

## Abstract

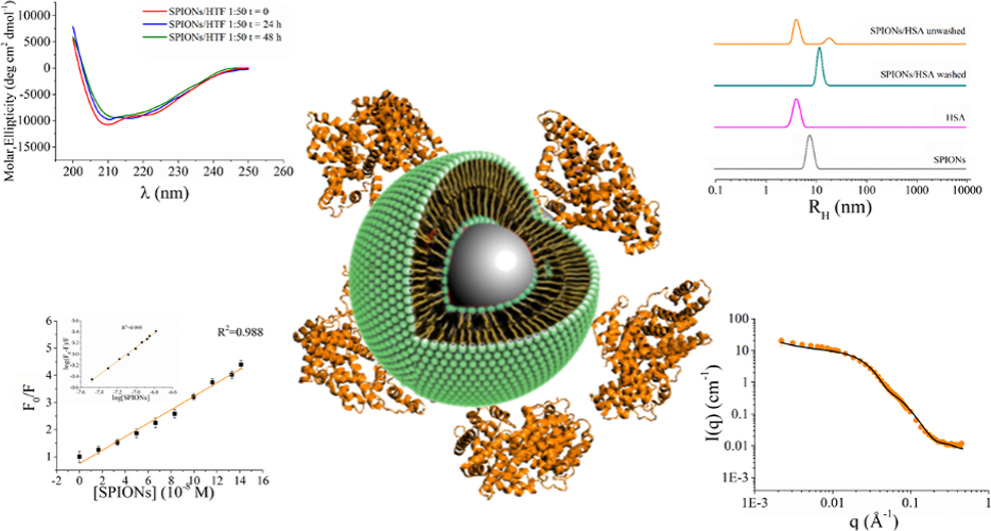

Nanoparticles (NPs) are increasingly exploited as diagnostic and therapeutic devices in medicine. Among them, superparamagnetic nanoparticles (SPIONs) represent very promising tools for magnetic resonance imaging, local heaters for hyperthermia, and nanoplatforms for multimodal imaging and theranostics. However, the use of NPs, including SPIONs, in medicine presents several issues: first, the encounter with the biological world and proteins in particular. Indeed, nanoparticles can suffer from protein adsorption, which can affect NP functionality and biocompatibility. In this respect, we have investigated the interaction of small SPIONs covered by an amphiphilic double layer of oleic acid/oleylamine and 1-octadecanoyl-*sn*-glycero-3-phosphocholine with two abundant human plasma proteins, human serum albumin (HSA) and human transferrin. By means of spectroscopic and scattering techniques, we analyzed the effect of SPIONs on protein structure and the binding affinities, and only found strong binding in the case of HSA. In no case did SPIONs alter the protein structure significantly. We structurally characterized HSA/SPIONs complexes by means of light and neutron scattering, highlighting the formation of a monolayer of protein molecules on the NP surface. Their interaction with lipid bilayers mimicking biological membranes was investigated by means of neutron reflectivity. We show that HSA/SPIONs do not affect lipid bilayer features and could be further exploited as a nanoplatform for future applications. Overall, our findings point toward a high biocompatibility of phosphocholine-decorated SPIONs and support their use in nanomedicine.

## Introduction

Small particles with dimensions less than 100 nm are known as nanoparticles (NPs).^1,2^ They are characterized by properties that are very different from those of both their constituent atoms/molecules and corresponding bulk materials because of their small size and large surface-to-volume ratio.^3^ These unique NP properties are size-dependent, thus being in principle prone to be properly tuned,^1^ and cover a wide range of phenomena making them powerful tools to be used in a variety of fields including energy,^4^ environment,^5^ electronics,^6^ biology,^7^ and biomedicine.^2,8−10^ Concerning the last point, recent years have seen numerous innovations in using nanoparticles for medical imaging, novel therapeutic approaches, and drug delivery.^8,9,11^ The reasons for the impressive development of nanoparticles in medicine are various: (i) as stated above, nanoparticles possess unique physical and chemical properties, such as optical, magnetic, electrical, and electro-optical properties that can be exploited for both diagnosis and therapy; (ii) they are small enough to interact directly with the cellular machinery and efficiently reach otherwise inaccessible targets; (iii) they are optimally suited to be transported in the bloodstream and achieve good clearance.^12,13^

Among the NPs widely studied for biomedical applications, there are superparamagnetic iron oxide nanoparticles (SPIONs).^9,14−17^ Probably the best-known application of SPIONs in nanomedicine is as magnetic resonance imaging (MRI) contrast agents^14−18^ and as local heaters for hyperthermia-based therapies.^19^ However, they can be also used as nanoplatforms for multimodal imaging, for example, by conjugation with optical imaging probes such as dye molecules, dye-doped silica materials, quantum dots, etc., or by adding a proper chelate molecule able to bind radioisotopes for PET applications,^20^ and as drug carriers, thus obtaining theranostic devices, realizing the need for real time monitoring of the therapy.^14,17,18^

Despite the great development of nanoparticles for biomedical uses, combining nanomaterials with biology presents several challenges.^21^ The first issue to take into account is the stark contrast between the organic solvents in which nanomaterials are often produced and the complexity of common biological fluids, which are aqueous solutions with high salt and very high biomolecule concentrations.^13^

The very first problem, i.e., facing the aqueous phase, can be overcome through a proper functionalization of the NP surface.^22^ In this respect, some of us have developed an easy and versatile functionalization protocol based on hydrophobic interaction between the first layer of molecules that cover the NP surface exposing their apolar tails, as deriving from the synthesis step (i.e., oleic acid, oleylamine), and proper amphiphiles, such as phospholipids (for example 1-octadecanoyl-*sn*-glycero-3-phosphocholine, 18LPC). The resulting NP will present a double layer of amphiphiles and will be soluble in water media. This strategy has been applied successfully to different metal NPs, such as SPIONs^23,24^ and Au NPs.^25^ Moreover, as additional advantages, this approach does not involve any purification step and allows different functionalization to be performed by adding proper amphiphilic molecules, for example, amphiphilic drugs^26,27^ or target agents.^28^

As said before, biological fluids are not only based on water and salts but also include thousands of different species, with a protein concentration greater than ∼300 mg mL^–1^. This is why biological environments often lead to unpredictable behavior of inorganic materials.^29^ Thus, the encounter with the complexity of the biological world, particularly proteins, is much more challenging than making NPs water-soluble. Nanomaterials can suffer from irreversible nonspecific adsorption and protein corona formation, the adsorption of proteins from the surrounding media onto the NP.^30−33^ This can cause protein denaturation that can result in toxic effects and/or NP aggregation,^34,35^ affect NP stability, induce aggregation with the loss of NP function and possible toxic effects,^36−38^ change the surface properties, and alter the targeting ability of the NP.^29,31,39^

In the last years, a great number of studies have investigated different aspects of the formation and the consequences and utilization of the protein-corona in specific nanoparticle–protein systems. In particular, these studies can be broadly divided into three classes: (1) those focusing on the effect of nanoparticles on protein structure and properties; (2) those analyzing the influence of protein molecules on nanoparticle stability and surface functionality; and (3) those investigating the behavior of nanoparticle–protein composites in biological contexts.^40^ In this respect, here we aim at presenting a thorough investigation of systems formed by SPIONs functionalized with 18LPC (hereafter simply SPIONs) and two abundant blood proteins, human transferrin (HTF) and human serum albumin (HSA). In particular, we analyzed the effects of SPIONs on the protein secondary and tertiary structures, as well as protein thermal stability, by means of circular dichroism and fluorescence spectroscopy. Based on results indicating a significant binding only in the case of HSA–SPIONs, further confirmed by fluorescence quenching experiments, for this system we extended the study to include the effects of HSA on the NP structure and stability, which were analyzed by means of dynamic and electrophoretic light scattering as well as small-angle neutron scattering. Finally, because one of the concerns about these nano-objects in biological fluids is the lack of knowledge about how they interact with biologically relevant interfaces, specifically cell membranes,^41^ we also investigated the behavior of SPIONs/HSA with respect to lipid bilayers mimicking biological membranes.

## Experimental Section

### Materials

For the synthesis of the nanoparticles, iron(III) acetylacetonate (Fe(acac)_3_, purity grade 99%), 1,2-hexadecanediol (90%), oleylamine (70%), oleic acid (99%), diphenyl ether (99%), ethanol (98%), and cyclohexane (≥99.9%) were used as received from Sigma-Aldrich. For the functionalization step, 1-stearoyl-2-hydroxy-3glicerno-*sn*-phosphocholine (18LPC > 99%) was purchased from AvantiPolar Lipids Inc. The proteins human serum albumin (HSA) and apo-human transferrin (HTF) were purchased from Sigma-Aldrich. HSA was fatty acid free and globulin free, with a purity grade ≥99%; HTF has a purity grade ≥98% and an iron content ≤0.005%.

### SPION Synthesis and Functionalization

SPIONs were synthesized as previously reported through a modified version of the thermal decomposition method:^42^ namely, iron(III) acetylacetonate, oleylamine, oleic acid, and diphenyl ether were mixed together in a three-neck flask, and the solution was heated at 100 °C under argon atmosphere with vigorous stirring. Then 1,2-hexadecanediol and another aliquot of diphenyl ether were added to the flask and kept at 200 °C for 30 min. For the last 90 min, the temperature was increased to 280 °C. At the end of the synthesis, the suspension was washed with cold ethanol and centrifuged at 6000 rpm for 20 min, twice. SPIONs are thus obtained as a dark brown solid precipitate, which is gradually dispersed in hexane. The iron concentration in the final product was evaluated by means of inductively coupled plasma mass spectrometry.^23,24,26^

By considering the nanoparticle dimension and Fe_3_O_4_ density, starting from the Fe concentration we evaluated an indicative nanoparticle molar concentration as described in the Supporting Information.

Nanoparticles were functionalized through a stratification protocol; i.e., the suspension of SPIONs in hexane was stratified over an aqueous solution of 1-octadecanoyl-*sn*-glycero-3-phosphocholine (18LPC) at a SPIONs/18LPC molar ratio of 1:1000 and sonicated in an ultrasound thermostat bath at 50 °C. After about 2 h, a clear brown water dispersion of nanoparticles, covered with a bilayer of oleic acid/oleylamine and 18LPC, is obtained.^23,24,26^ The resulting functionalized nanoparticle solutions have a concentration of about 1 mg mL^–1^, expressed as Fe concentration, corresponding to about 10^–6^ M NP concentration. Hereafter nanoparticles coated with 18LPC are simply called SPIONs.

SPIONs were extensively characterized by some of us,^23,24,26^ while here we have reported DLS and ELS analysis for comparison with NP–protein systems.

### Protein–SPIONs Samples

Stock solutions of protein have been prepared by dissolving them in either phosphate-buffered saline solution (PBS) or 10 mM sodium phosphate buffer at pH 7.4. Each protein sample was filtered on a 0.2 μm filter in order to remove the impurities, and then the protein concentration was determined by means of UV spectroscopy at 280 nm. Protein–SPIONs samples were prepared by proper mixing of stock solutions in order to achieve the desired NP–protein molar ratio, 1:50, 1:100, in order to mimic the average concentration ratio expected to be used in *in vivo* applications,^26^ and the protein concentration necessary for the experimental technique used, or the increasing ratios required by fluorescence quenching experiments. Resulting solutions were either used with no further treatment or extensively washed to remove excess protein. In the latter case we used a Centricon mini-concentrator with a vertical membrane and cutoff of 100 kDa (Millipore) and performed several washing steps by centrifugation at 20 °C and 10000*g*. At each step, the washing waters were removed from the bottom of the Centricon, and an equal volume of buffer solution was added to the protein/NP sample in the Centricon in order to keep the concentration of the sample constant.

### Circular Dichroism

Circular dichroism (CD) spectra were recorded at 20 °C using a Jasco J-715 spectropolarimeter equipped with a Peltier thermostatic cell holder (Model PTC-348WI). CD measurements were carried out in the 260–200 nm range, using a 0.1 cm path length cell and solutions at 0.2 mg mL^–1^ protein concentration in either PBS or 10 mM Na-phosphate buffer at pH 7, with 0.5 nm data pitch, 2 nm bandwidth, and 20 nm min^–1^ scanning speed. The same conditions were employed in the case of both free proteins and proteins incubated with SPIONs. Thermal unfolding curves were obtained by following the CD signal at 222 nm in the 10–90 °C range, at a heating rate of 1.0 °C min^–1^.

The unfolded fraction was calculated as
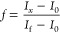
1where *f* is the denatured fraction, *I*_*x*_ is the CD intensity at 222 nm at the temperature *T*, and *I*_0_ and *I*_f_ are the intensity at the lowest temperature and at the highest temperature, respectively. We plotted *f* as a function of *T* and calculated the melting temperatures by fitting the experimental data with a sigmoidal function.

### Fluorescence Spectroscopy

Fluorescence spectra were recorded at 20 °C using a Horiba Scientific Fluoromax-4 spectrofluorometer equipped with a Peltier control system and 1 cm path length cells. The same solutions at 0.2 mg mL^–1^ protein concentration and 1:50 or 1:100 NP:protein ratio that were analyzed by means of CD spectroscopy were employed for fluorescence spectroscopy. Tryptophan residues were selectively excited at 295 nm, whereas both tyrosine and tryptophan residues were excited at 280 nm; in the latter case the investigated emission range was 295–500 nm, and in the former 310–500 nm. Both excitation and emission slit widths were set to 5 nm.

The interaction between proteins and SPIONs was monitored by means of fluorescence quenching experiments, by preparing nanoparticle–protein solutions at fixed protein concentration, optimized to have the maximum fluorescence intensity, and increasing the nanoparticle concentration in the nanoparticle–protein molar ratio 1:100–1:20 range with a 10-fold increase of NP content from one sample to the other; i.e., we analyzed samples with 1:100, 1:90, 1:80, and so on protein–NP molar ratios. Each solution was incubated for 2 h before the measurements.

The first analysis of quenching data was performed by means of a Stern–Volmer plot: the ratio between the fluorescence intensity in the absence of quencher, *F*_0_, and that in the presence of quencher, *F*, was plotted as a function of the quencher (i.e., SPIONs) concentration. In the case of HSA, a straight line was obtained, allowing the Stern–Volmer constant *K*_SV_ to be determined through
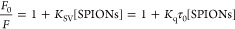
2where τ_0_ is the mean fluorescence lifetime of the fluorophore (HSA) and *K*_q_ is the quenching efficiency. The calculated *K*_q_ value allowed us to distinguish between static and dynamic quenching phenomena, with the former characterized by *K*_q_ greater than and the latter smaller than 2 × 10^10^ M^–1^ s^–1^.^29,43^

In the presence of static quenching, data can be also treated by modified Stern–Volmer analysis by plotting  as a function of log[quencher] and employing
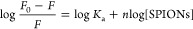
3It is possible to determine the binding constant, *K*_a_, from the intercept, and the number of quencher binding sites, *n*, from the slope of the straight line.^29,44^

### Dynamic Light Scattering

The dimensions of SPIONs/protein assemblies, and of isolated proteins and nanoparticles for comparison, as well as the possible formation of aggregates were evaluated by dynamic light scattering (DLS). DLS measurements were performed on a homemade instrument composed of a Photocor compact goniometer, a SMD 6000 Laser Quantum 50 mW light source operating at 5325 Å, a photomultiplier (PMT-120-OP/B), and a correlator (Flex02-01D) from Correlator.com. The experiments were carried out at 25.0 °C, keeping the temperature constant by a thermostatic bath, and at a scattering angle θ = 90°. For DLS analysis 10 or 20 mg mL^–1^ protein samples were used, while SPIONs concentration was about 1 mg mL^–1^. The scattered intensity correlation function was analyzed using a regularization algorithm.^45^ The diffusion coefficient of each population of diffusing particles was calculated as the Z-average of the diffusion coefficients of the corresponding distributions. All the samples being diluted solutions, the Stokes–Einstein equation was used to evaluate the hydrodynamic radius, *R*_H_, from translation diffusion coefficients, *D*.^45^

### Electrophoretic Light Scattering

Surface charge (zeta potential) of SPIONs, proteins, and SPIONs/protein assemblies was evaluated by means of electrophoretic light scattering using a Zetasizer Nano ZSP (Malvern Instruments, England). All the measurements were performed using 10 mg mL^–1^ protein or protein/SPION solutions and 1 mg mL^–1^ SPION solutions in 10 mM sodium phosphate buffer pH 7.4, previously filtered with 220 nm cutoff microfilters, and polystyrene Folded Capillary Zeta cells (Malvern Instruments). Each measurement was performed at 25 °C upon a 30 s equilibration time, and the average of three measurements at a stationary level was taken. The zeta potential was calculated by applying the Smoluchowski model.

### Small-Angle Neutron Scattering

Small-angle neutron scattering (SANS) measurements were performed at 25 °C on the KWS-2 diffractometer operated by Julich Centre for Neutron Science at the Heinz Maier Leibnitz Zentrum, Garching (Germany).^46−48^ For both HSA (30 mg mL^–1^ protein concentration) and SPIONs/HSA (10 mg mL^–1^ NP concentration incubated with HSA in a 1:50 NP:protein ratio and then purified to remove protein excess) samples, an incident wavelength of 5 Å and wavelength spread of Δλ/λ ≤ 0.1 were used. A two-dimensional array detector at three different wavelength (*W*)/collimation (*C*)/sample-to-detector (*D*) distance combinations (*W* 5 Å/*C* 8 m/*D* 2 m; *W* 5 Å/*C* 8 m/*D* 8 m; *W*5 Å/*C* 20 m/*D* 20 m) measured neutrons scattered from the samples. These configurations covered a *q* range from 0.08 to 0.4 Å^–1^. The raw data were corrected for background and empty cell scattering. The absolute scattering cross section dΣ/dΩ data were plotted as a function of the scattering vector *q* obtaining a scattering profile. The dependence of dΣ/dΩ on the scattering vector can be summarized as
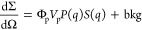
4where ϕ_p_ and *V*_p_ represent the volume fraction of the particles and the particle volume, respectively, *P*(*q*) and *S*(*q*) are the form and the structure factor of the scattering particles, and bkg is the incoherent and inelastic part of the scattered cross section, largely dependent on any hydrogen present. The form factor is responsible for the shape, size, and size distribution of the scattering particles, while a contribution of the structure factor can be considered when an interparticle correlation exists. Experimental data were fitted with an appropriate model by using the SASview program^49^ in order to get structural information contained in the form factor.

### Neutron Reflectivity

Neutron reflectivity experiments aimed at investigating the interaction between model membranes and SPIONs/HSA were performed at 25 °C with the MARIA Reflectometer operated at the Julich Centre for Neutron Science at the Heinz Maier Leibnitz Zentrum, Garching (Germany)^50^ and the D17 reflectometer operated at the Institute Laue Langevin (ILL), Grenoble, France.^51,52^

We used bilayers with two different lipid compositions, namely, 1-palmitoyl-2-oleoyl-*sn*-glycero-3-phosphocholine/1-palmitoyl-2-oleoyl-*sn*-glycero-3-(phospho-rac-(1-glycerol))/cholesterol (POPC/POPG/Chol) 72:8:20 and 54:6:40 mol/mol/mol as model membranes. Each bilayer was characterized before and after the addition of either SPIONs/HSA solutions at 1 mg/mL iron concentration or HSA solution, in three different contrast media, H_2_O, D_2_O, and silicon match water (SMW). NR profiles were fitted to a box model using the Aurore software^53^ and characterizing each box by its thickness, scattering length density (sld), solvent volume fraction, and interfacial roughness. The first two boxes correspond to the silicon block and the thin solvent layer interposed between it and the supported bilayer; the remaining three boxes describe the lipid bilayer, namely, the inner headgroups, the hydrophobic chains, and the outer headgroups. In the case of systems containing HSA or SPIONs/HSA, an additional layer was taken into account.

## Results and Discussion

### Effect of NPs on the HTF and HSA Structure

**Figure 1:**
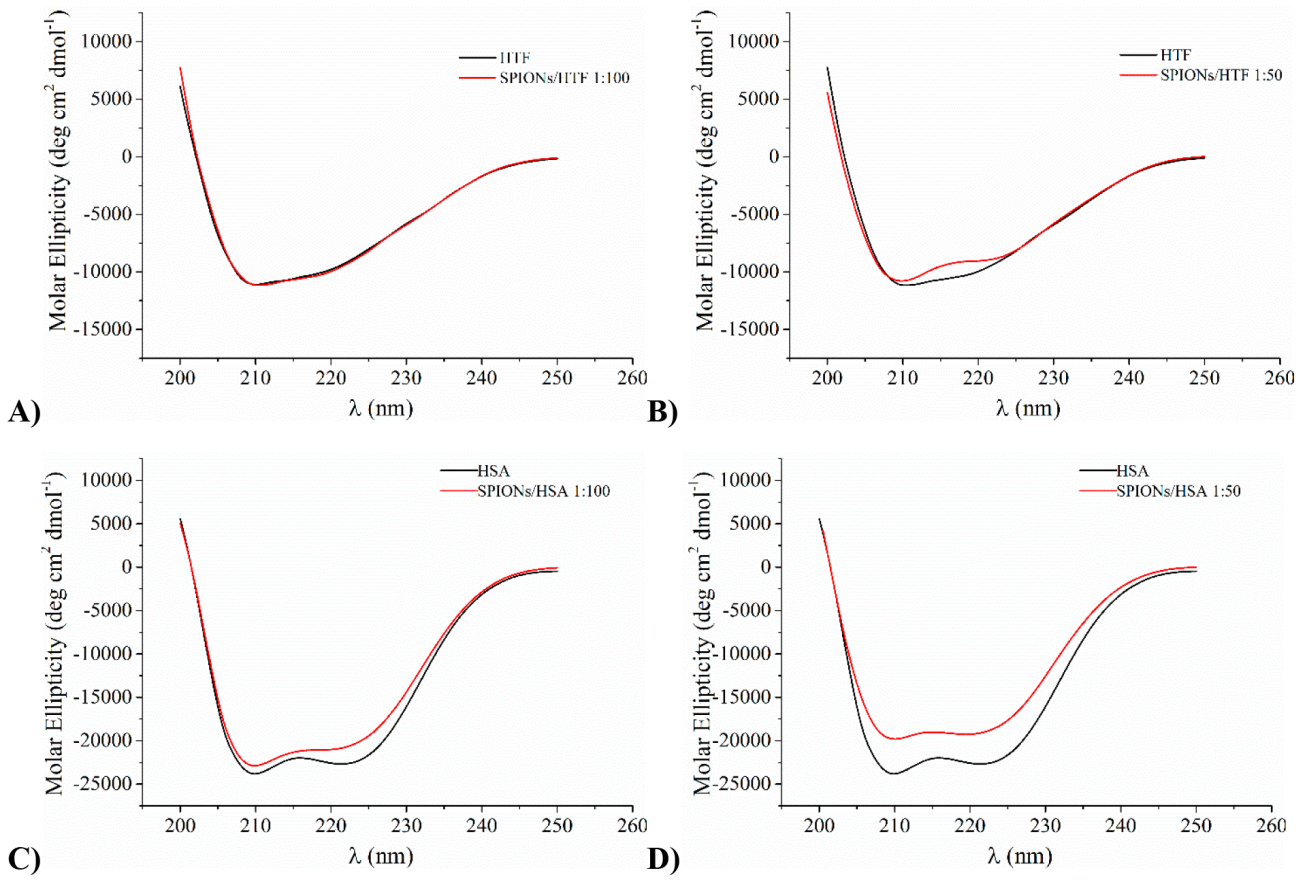
Comparison of far-UV CD spectra of HTF (panels A and B) and HSA (panels C and D) incubated in the presence of SPIONs at two different NP:protein molar ratios (1:100, panels A and C; 1:50, panels B and D) with respect to spectra of pure proteins.

The first indication of the effect of NPs on serum proteins human transferrin (HTF) and human serum albumin (HSA) was obtained by comparing the CD spectra of HTF and HSA incubated with NPs in two different NP:protein molar ratios, 1:100 and 1:50, with those of the corresponding pure protein in PBS (Figure 1). This is possible because the CD spectra of SPIONs are featureless and with near-zero intensity (data not shown); thus, they do not affect the CD spectra of proteins. NP:protein samples were used with no further purification, i.e., in the presence of excess protein. In the case of NP:HTF 1:100, the spectrum is completely superimposable with that of pure protein, with two minima at about 208 and 222 nm and a maximum below 200 nm (Figure 1A), in good agreement with the predominantly α-helicoidal structure of this protein.^54^ At the higher NP:protein ratio, only a very slight change of intensity is observed (Figure 1B), pointing toward no significant change of the transferrin secondary structure induced by the presence of NPs.

In the case of HSA, a slight change of the CD spectrum is observed in the NP:HSA 1:100 system; in particular, the minimum at λ ≈ 222 nm is less intense than that found for pure HSA (Figure 1C). At the NP:protein 1:50 ratio, the intensity of the CD spectrum further decreases with respect to the pure protein, but the shape is essentially the same (Figure 1D). These findings point toward an interaction between human serum albumin and NPs. Moreover, a slight change in CD may arise from protein molecules that have been sitting on the NP surface and desorb with slightly different conformation.

However, even affecting protein structure NPs do not cause unfolding or dramatic changes of the secondary structure.

**Figure 2:**
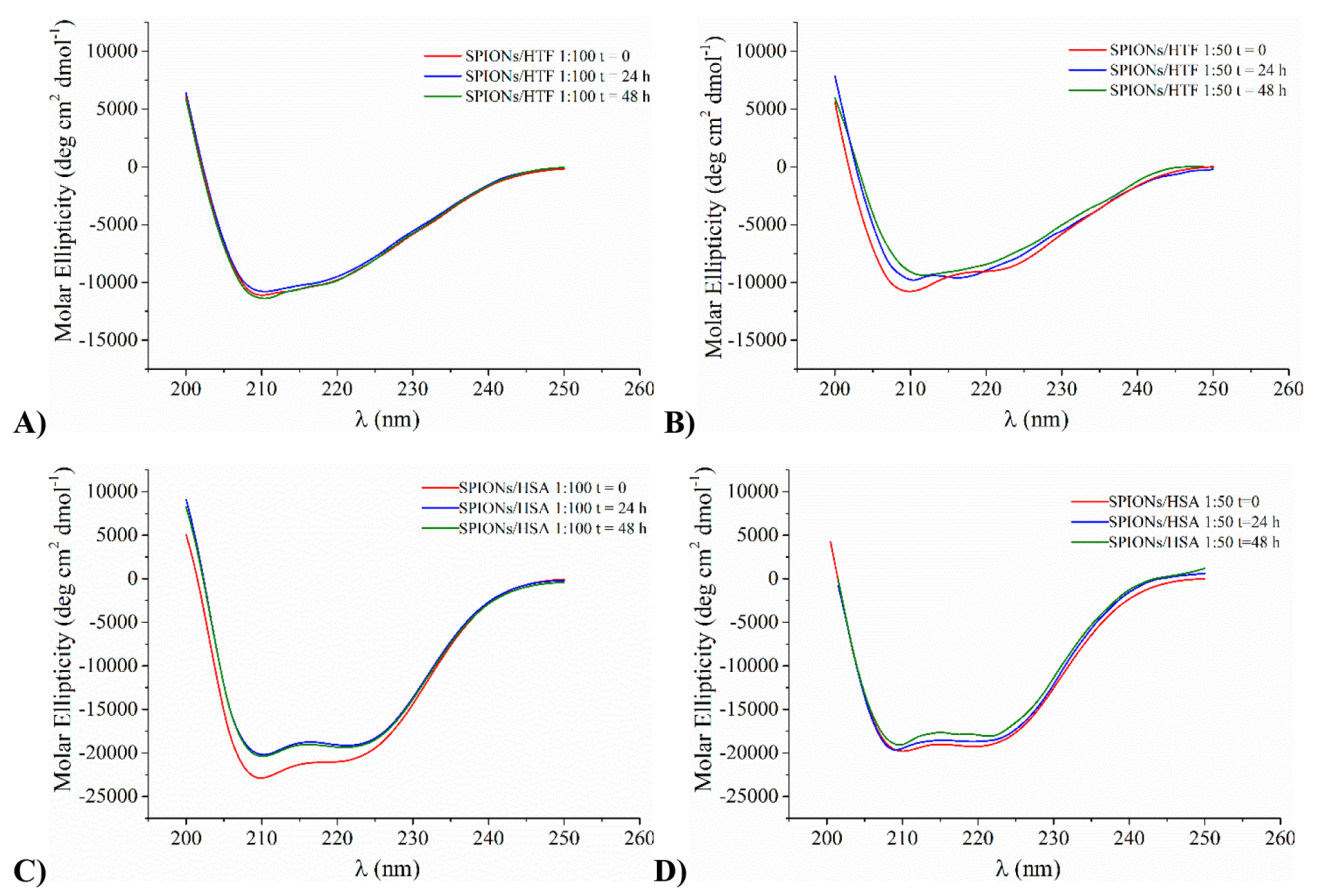
Time evolution of far-UV CD spectra of HTF (panels A and B) and HSA (panels C and D) incubated in the presence of SPIONs at two different NP:protein molar ratios (1:100, panels A and C; 1:50, panels B and D) during 2 days observation.

All NP–protein samples were analyzed for 48 h, with the aim at revealing any change induced by long incubation times. In the case of HTF, no change is observed at both 1:50 and 1:100 ratios (Figure 2 A and B), while for HSA, at both NP:protein ratios, a slight intensity decrease occurs in the first 24 h, but no further change was observed in the following hours (Figure 2 C and D). Significantly, no precipitation or change in the appearance of the solution was detected in any case.

**Figure 3:**
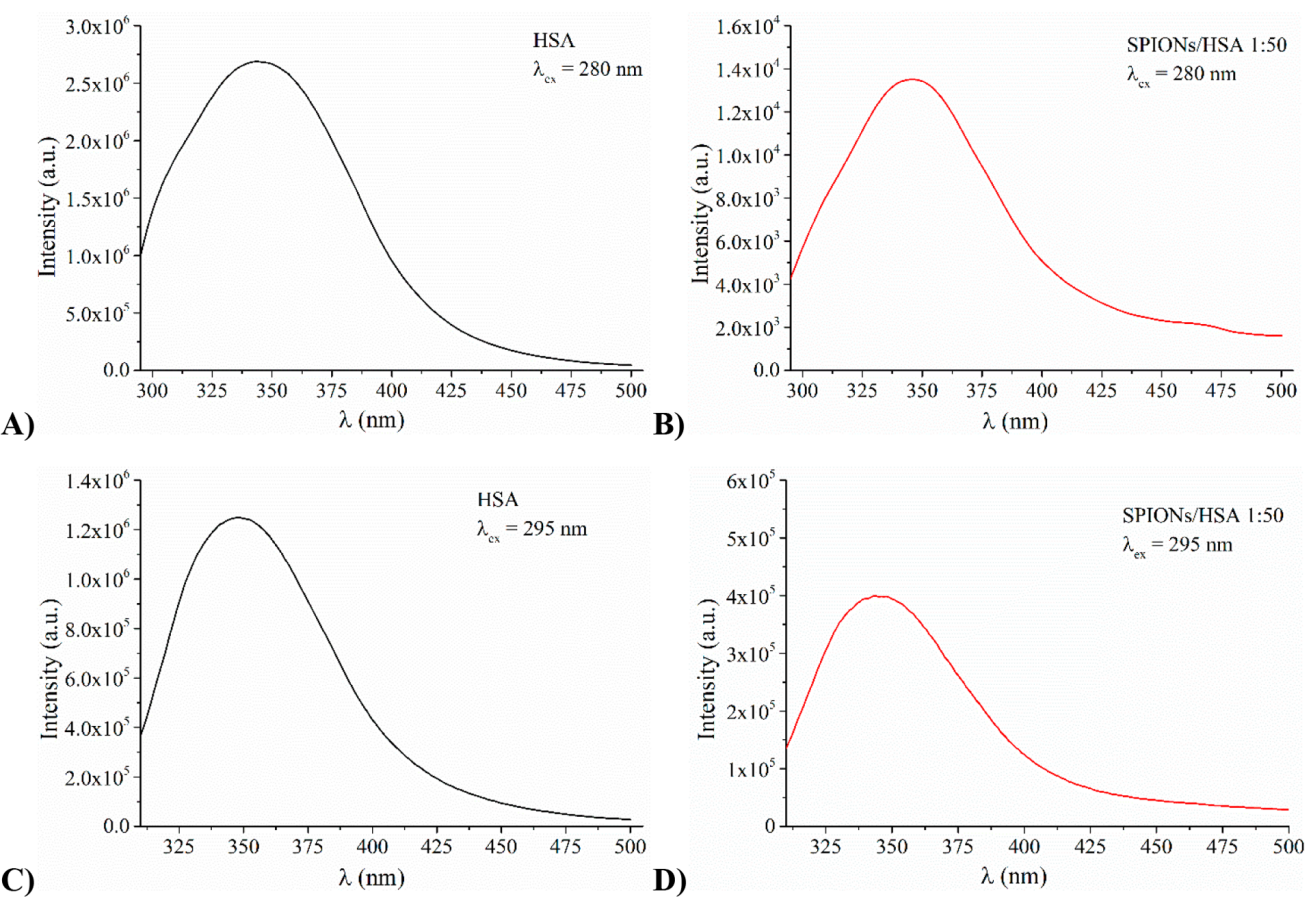
Fluorescence spectra of HSA in the absence (panel A and C) and in the presence of SPIONs at NP:protein molar ratio 1:50 (panel B and D) obtained by exciting at either λ_ex_ = 280 nm (panels A and B) or λ_ex_ = 295 nm (panels C and D).

The effects of the NP on the protein tertiary structure were investigated by means of fluorescence spectroscopy for samples with the NP:protein molar ratio 1:100 or 1:50. In the former case, no change is observed with the exception of a decrease in fluorescence intensity with both HTF and HSA (data not shown), with SPIONs acting as quenchers of protein fluorescence. At the NP:protein molar ratio 1:50, in the case of HTF the presence of the NP does not induce any shift of the emission maximum, either with excitation wavelength 280 nor 295 nm (Figure S1), indicating that the environment of aromatic residues is not affected at all by the presence of NPs. In the case of HSA, no shift is observed for spectra obtained with excitation wavelength 280 nm, while a blue shift (about 4 nm) is found with λ_ex_ = 295 nm (Figure 3), indicating that the only tryptophan residue of HSA experiences a more apolar environment with respect to HSA alone, probably due to the interaction with NPs.

At the 1:50 NP:protein ratio, the quenching of protein fluorescence is even more marked (see also Figure S2 where the spectra of Figure 3 have been overlapped) suggesting a NP concentration-dependent phenomenon.

The very small change of fluorescence spectra does not seem consistent with any change of protein tertiary structure: SPIONs do not cause a significant change of protein secondary and tertiary structure for both HTF and HSA.

Interestingly, when incubated in 18LPC solutions, a significant blue shift of the maximum fluorescence wavelength occurs in SPIONs/HSA systems (Figure S3). An about 10 nm a blue shift in the case of excitation at either 280 or 295 nm indicates that aromatic residues experience a more apolar environment, likely due to extensive binding of 18LPC, which should be in part hindered when it is closely packed on the SPION surface.

### Effect of NPs on HTF and HSA Structure Stability

With the aim of investigating whether NPs can alter protein stability and assessing a HTF–NP interaction not immediately evident from the comparison of CD spectra, we performed thermal unfolding experiments for different samples and compared melting profiles and melting temperatures with those of pure proteins. In particular, the CD signal at 222 nm was followed in the 10–90 °C range, and the corresponding CD signal was used to calculate the unfolded fraction.

**Figure 4:**
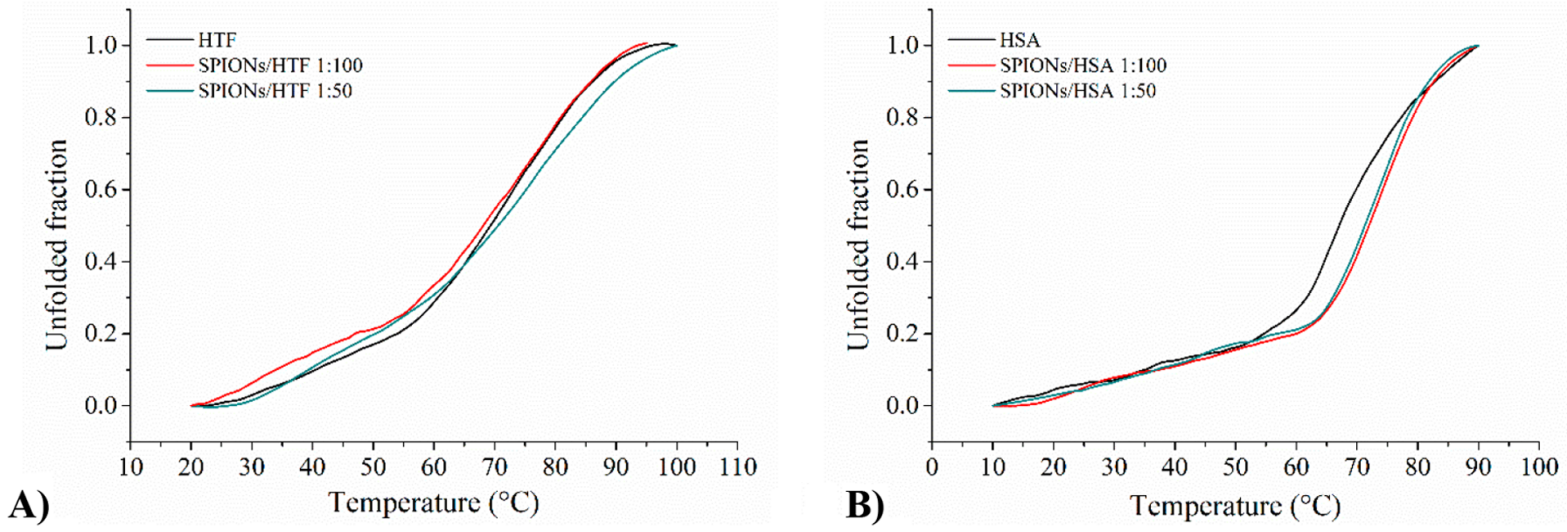
Melting profiles of HTF (panel A) and HSA (panel B) alone and incubated in the presence of SPIONs at 1:100 and 1:50 NP:protein molar ratios, as derived from the analysis of CD spectra in the 10–90 °C temperature range.

Melting profiles for HTF and HSA, alone and incubated with NP at two different NP:protein molar ratios, are reported in Figure 4 A and B, respectively. All melting profiles present a sigmoidal shape that is not affected by the presence of NPs, indicating a mostly cooperative unfolding. In the case of HTF the three curves are perfectly superimposable, with no effect of NPs; in contrast, in the case of HSA a slight shift toward higher temperatures is observed for both NP:HSA systems, with *T*_m_ = 69 and 73 °C for HSA and NP:HSA, respectively.

No melting profile change was observed in the following 2 days (as an example, see SPIONs/HSA 1:50 in Supporting Information Figure S4).

Overall CD analysis points toward a NP:HSA interaction, resulting in a slight change of protein structure, which is more stable with respect to temperature than native HSA structure. In contrast, no indication of a HTF–NP interaction arises: either NPs do not affect any of the HTF properties or they do not interact at all.

When CD spectra and thermal profiles of HSA in the presence of 18LPC and HSA alone are compared (Figure S5), no significant difference is observed. Thus, even if some residual 18LPC were present in nanoparticle samples, the different behavior of the protein in the presence of NPs and in the presence of 18LPC seems to indicate that HSA interacts mainly with NPs in these samples.

### Insights into SPIONs/Protein Recognition

Fluorescence spectra of proteins in the presence of SPIONs have revealed that nanoparticles act as quenchers of protein fluorescence, and this behavior can be used to get information on the binding process, in particular to estimate the binding constant. Indeed fluorescence quenching was shown to be a convenient method to investigate NP–protein interactions, giving results consistent with those obtained by ITC,^30^ and this is particularly true for protein containing exposed aromatic residues. HSA has only one Trp, not buried in the inner core of the protein but located in a superficial pocket accessible to the solvent, as highlighted by the λ_max_ = 348 nm that is about 15 nm red-shifted with respect to typical values of buried Trp residues, and to ligand binding.

**Figure 5:**
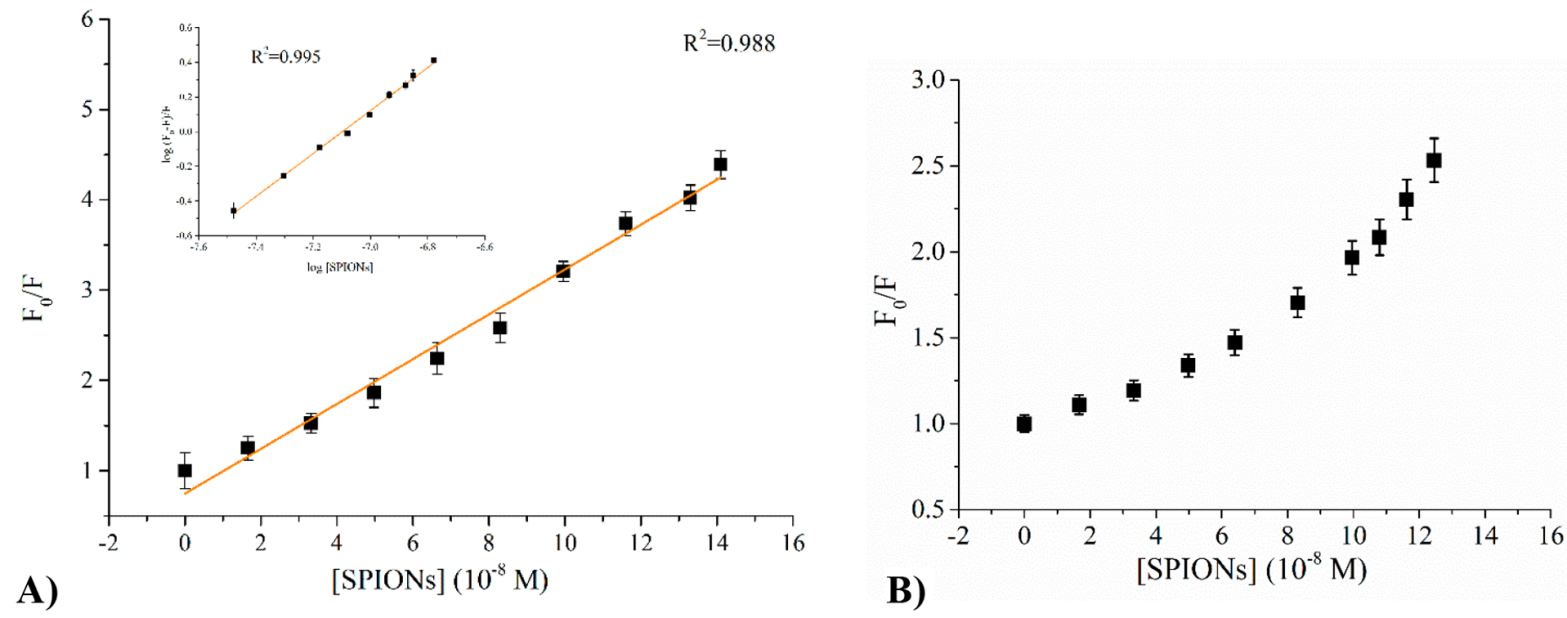
Stern–Volmer plots determined for SPIONs/HSA (A) and SPIONs/HTF (B) systems at fixed protein concentration and increasing SPION concentration. λ_exc_ = 295 nm. For SPIONs/HSA, the modified Stern–Volmer plot is reported in the inset of panel A.

We acquired fluorescence spectra at fixed protein concentration and increasing SPION concentration (Figure S6). Data were elaborated through the Stern–Volmer analysis, by plotting the ratio between the protein fluorescence intensity with no quencher (*F*_0_) and the observed fluorescence in the presence of a quencher (*F*) as a function of the quencher concentration. In the case of HSA, experimental data fall on a straight line (Figure 5A) that was fitted according to eq 2 above.

From the slope we determined a Stern–Volmer constant value *K*_SV_ = 2.5 × 10^7^ M^–1^, by knowing the mean fluorescence lifetime of HSA, τ_0_ = 7.1 × 10^–9^ s, as reported in the literature,^55^ a quenching efficiency *K*_q_ = 3.8 × 10^15^ M^–1^ s^–1^ results. Quenching mechanisms can be distinguished for static and dynamic depending on the *K*_q_ value. The static mechanism implies the formation of a protein–quencher complex stabilized by a strong interaction, whereas dynamic quenching implies a weak interaction between the protein and the quencher. The *K*_q_ value (3.8 × 10^15^ M^–1^ s^–1^) acquired here is exceptionally larger than the maximum diffusion-controlled bimolecular rate constant, and such observation indicates that we can confidently consider a static quenching occurring,^56,57^ pointing toward the formation of a stable HSA/SPION complex.

Being in the presence of static quenching, we analyzed data through a modified Stern–Volmer plot, by reporting  as a function of log[SPIONs] (inset of Figure 5A) in order to calculate the binding constant *K*_a_ and the number of binding sites on the fluorophore (i.e., HSA) *n*, by means of eq 3 above. We determined *n* = 1.2, indicating that there is only one kind of binding site for HSA binding on the SPION surface. Concerning the binding constant, we determine *K*_a_ = 6.2 × 10^8^ M^–1^. This value is higher than that found for binding of bovine serum albumin, a protein highly homologous to HSA to other SPIONs with different coating layers,^29,58^ confirming the importance of the coating molecules in determining NP properties.

In the case of HTF, the plot of *F*_0_/*F* as a function of SPION concentration does not describe a linear trend but has an exponential growth (Figure 5B), which is an index of the likely coexistence of both static and dynamic quenching phenomena. Thus, it can be inferred that a slight interaction between SPIONs and HTF occurs, but not a significant one as that observed for the HSA system. The interaction between NP and HTF is corroborated by dynamic light scattering analyses of SPIONs/HTF 1:100 and comparison with reference systems, i.e., isolated SPIONs and HTF. Isolated systems present a single peak indicating a monodispersed solution, with *R*_H_ equal to 8 ± 1 and 5 ± 1 nm for SPIONs and HTF, respectively. On the other hand, the SPIONs/HTF 1:100 system always presents a population due to the protein excess and a larger population, whose dimension changes from sample to sample and ranges between 20 and 40 nm (see Figure S7A). In the following 2 days of observation, large polydisperse aggregates formed (Figure S7B) as a result of further protein-mediated interactions or depletion effects.

### Characterization of SPIONs/HSA Assemblies

To get further insight into SPIONs/HSA assemblies, in particular on their nature and stability, considering the strong binding between the protein and the NP surface highlighted by the *K*_a_ value, we also analyzed SPIONs/HSA samples where the excess protein was washed away by using a Centricon mini-concentrator with 100 kDa cutoff. Once excess HSA was washed away, the SPIONs/HSA samples were analyzed by means of circular dichroism, dynamic and electrophoretic light scattering, as well as small-angle neutron scattering.

**Figure 6:**
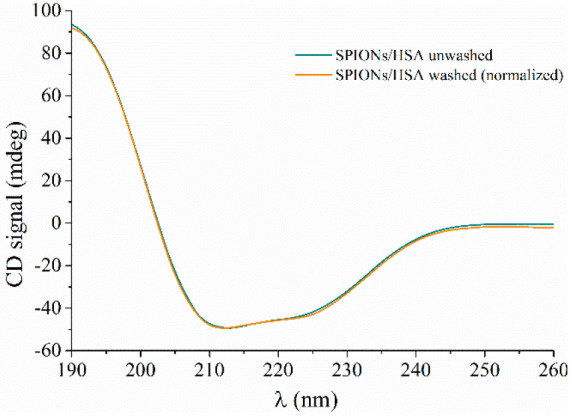
Comparison of normalized CD spectra for the SPIONs/HSA system before (unwashed) and after (washed) the removal of protein excess.

After extensive washing, the CD spectra of NP/protein samples show a marked decrease of overall intensity, coherently with a reduction of protein concentration due to the removal of HSA excess (Figure S8). However, they retain all the spectral features of HSA, i.e., the double minima at 222 and 208 nm and the maximum at 190 nm, and once normalized they are fully superimposable with those of the same systems before washing (Figure 6) (uncertainty on protein concentration does not allow a straight comparison of spectra without normalization). These findings prove not only that a significant fraction of HSA molecules is stably bound on NP surface but also that they are indeed well-structured folded protein molecules.

As a control, when the same procedure was applied to SPIONs/HTF samples, almost the entire protein was recovered in the washings since the CD spectra of washed SPIONs/HTF were featureless (data not shown), proving that HTF does not firmly bind NPs.

**Figure 7:**
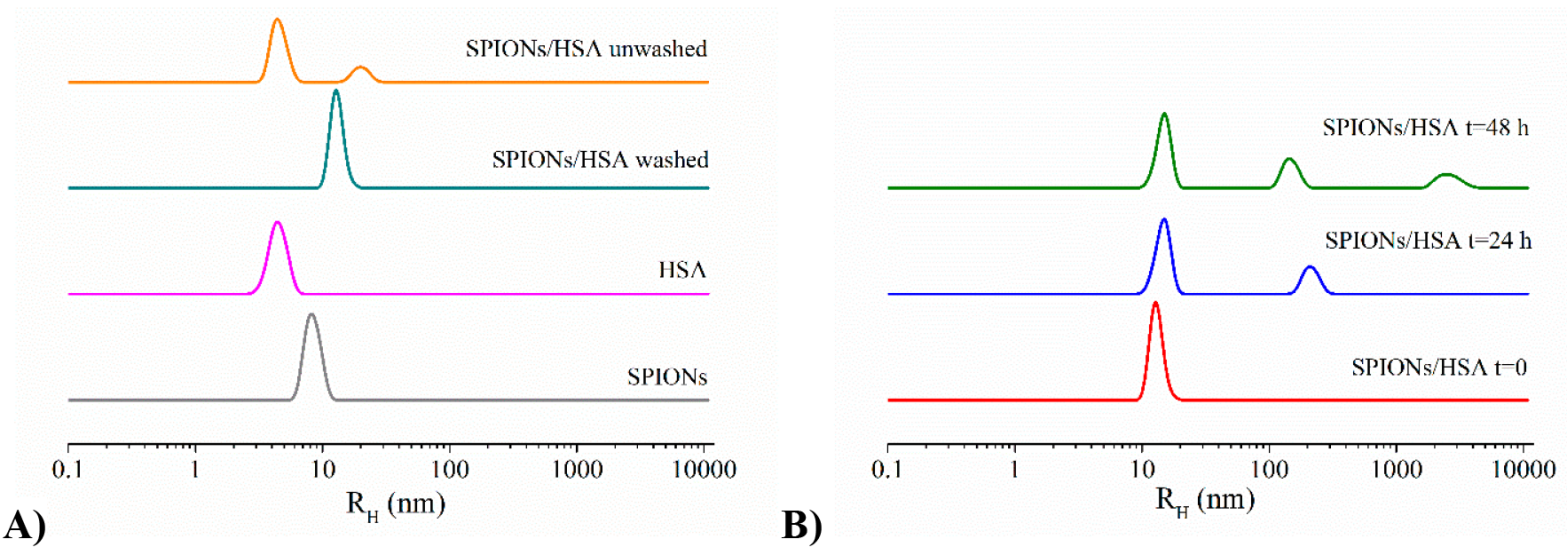
DLS profiles of SPIONs/HSA with respect to isolated SPIONs and protein (panel A); time evolution of DLS profiles of SPIONs/HSA during 2 days observation (panel B).

SPIONs/HSA systems were further characterized by means of dynamic light scattering. The determination of the hydrodynamic radius (*R*_H_) of SPIONs in the presence of HSA allows the formation of either a protein corona or NP–protein aggregates to be detected. With this aim, we characterized the different systems, the isolated protein and nanoparticles, and the protein/nanoparticles system. The analysis of isolated HSA shows the presence of a monodispersed species with *R*_H_ 4 ± 1 nm, and, similarly to what happens to SPION samples, it does not change with time. In the washed SPIONs/HSA system, upon removal of excess HSA, a single main population is present, characterized by a hydrodynamic radius higher than those of the isolated protein and nanoparticles (*R*_H_ = 11 ± 1) (Figure 7A). It is interesting that DLS analysis of SPIONs/HSA 1:100 before washing highlights the presence not only of the excess protein but also of a population larger than that observed in the washed system with *R*_H_ = 25 ± 3 nm. This population may be due to SPIONs/HSA aggregates or to formation of multiple HSA layers on the NP surface. The latter hypothesis should indicate a behavior different from that of BSA and other SPIONs.^29^ However, even considering the possible binding of HSA in multiple layers, only the first one is firmly anchored on the NP surface and is not removed by washing, suggesting formation of a hard and a soft corona of HSA.

In the following 2 days, we observe the formation of new populations: the main population can always be ascribed to the mixed NP/protein aggregates, even if larger aggregates are also visible (Figure 7B).

**Figure 8:**
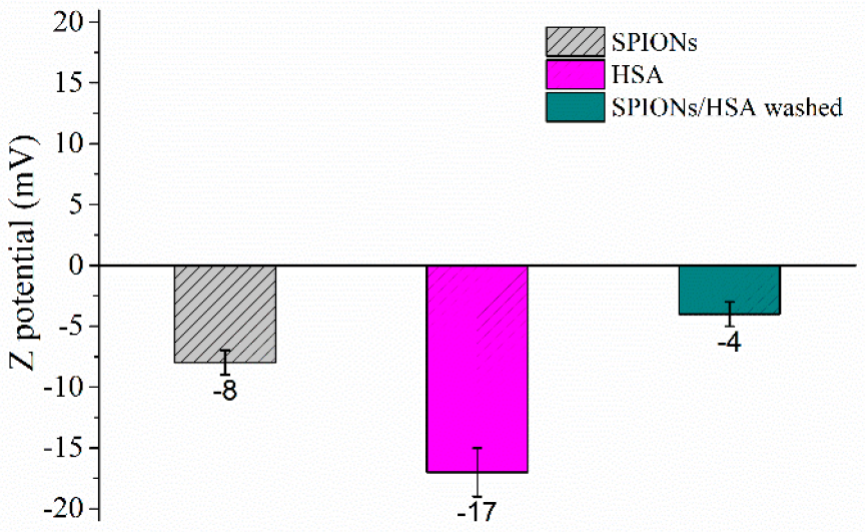
Comparison of zeta potential values for SPIONs/HSA after the removal of protein excess with respect to those of isolated SPIONs and protein.

The SPIONs/HSA system was characterized by means of electrophoretic light scattering to determine the surface charge of the aggregates in terms of Z potential. Measurements were performed in phosphate buffer, in order to avoid any interference due to the salts present in PBS. Before the analysis, CD spectra were recorded to assess that the change of buffer does not alter protein structural features (data not shown). For comparison, we analyzed isolated components as well (Figure 8).

SPIONs and HSA are both characterized by negative values of Z potential, indicating that they have the same net charge. However, several studies showed that the binding to negatively charged NP is not enhanced for proteins that were overall positively charged at pH 7.4. This happens because considering the Debye length at the typical ionic strengths of PBS buffer (and biological fluids), which is <1 nm and thus smaller than the size of a protein, Coulombic interactions are essentially only effective between charges located on the NP and protein surfaces that are in close contact.^59^ Patches with different net charge are indeed present on the HSA surface, and they may mediate its interaction with the SPION surface, as reported for other NP/protein systems.^60−62^ SPIONs/HSA also present a negative Z potential, but with a completely different value with respect to isolated components; in particular we observe a drop of zeta potential. This decrease can be attributed to the screening of NP and protein negative charges due to their reciprocal interaction.

Incidentally, it should be noted that the low absolute value of Z potential determined for the SPIONs/HSA system may be an index of a reduced stability of the species in solution, which in turn justifies the formation of the big aggregates observed in DLS profiles at long incubation times as some sort of aggregated SPIONs coated with HSA.

Finally, aimed at getting a deeper insight into the structural features of SPIONs/HSA aggregates, we employed SANS, which allows us to determine the shape of these aggregates and the thickness of the protein layer that likely coats the NPs.

**Figure 9:**
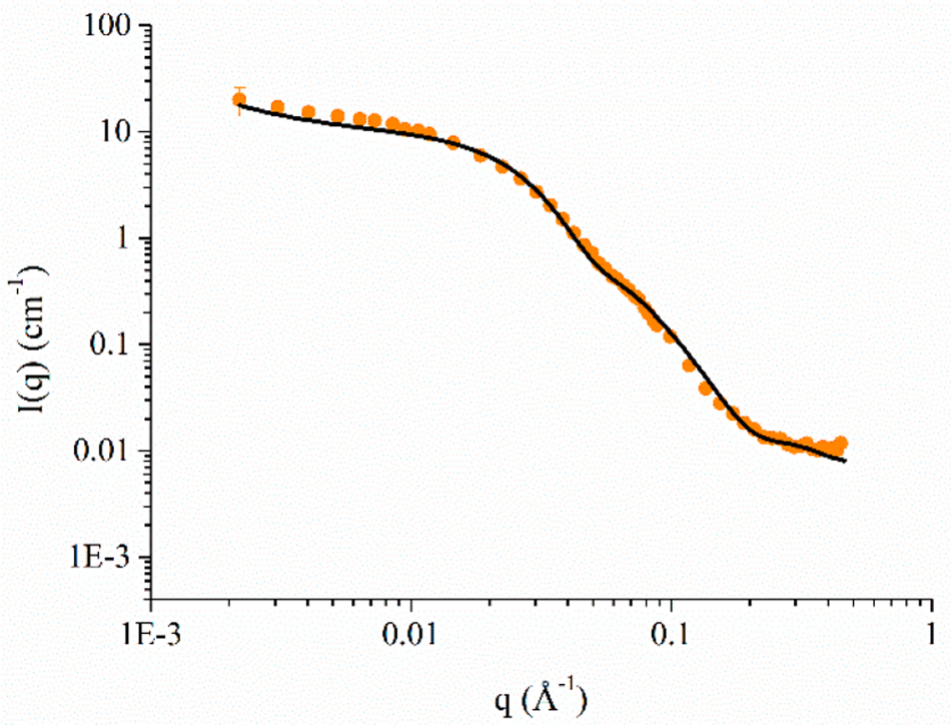
SANS profile of SPIONs/HSA upon the removal of protein excess. Best fitting curve is also shown in black.

The SANS profile of SPIONs/HSA upon the removal of excess protein is reported in Figure 9 (SANS profile of HSA is reported in Figure S9. Details on data fitting are in the Supporting Information). This scattering intensity profile is typical of nanoparticles, where the scattering length densities (sld) of the core and the solvent are almost matched. Furthermore, the system is characterized by some polydispersity that smooths oscillation in the scattering profiles. The slight slope of *q*^–1^ at low *q* suggests small nanoparticle clustering, which could be expected since the sample was analyzed a few days after purification and DLS already showed aggregate formation with time (Figure 7B). Experimental data were fitted using a core–shell sphere model (black line in Figure 9), with the Fe_3_O_4_ representing the core and the organic layers, including oleic acid/oleylamine, 18LPC, and the newly added protein, representing the shell, and a power law to take into account the rise at low *q* due to the presence of aggregated species. A Shultz polydispersity of the size of the NPs has been taken into account, resulting in a polydispersity index of about 0.6. The absence of any peak in the scattering profile indicates that the nanoparticles can be considered as noninteracting objects and a structure factor does not need to be included in the fitting procedure.

For comparison, the structural parameters obtained from the fitting of SPIONs/HSA, SPIONs, and HSA are reported in Table 1. What emerges is that the core features are not affected by the addition of HSA, as expected, with similar radius and sld values obtained. Significant differences arise when the shells of the two systems are compared: the shell sld value is 1 order of magnitude higher in the case SPIONs/HSA with respect to SPIONs; moreover, in the case of SPIONs/HSA the shell is thicker than that of SPIONs (48 vs 36 Å, respectively). The thicker shell and the sld value comparable with that of HSA suggests the presence of HSA on the NP surface.

**Table 1:** Structural Parameters for SPIONs/HSA in Comparison with Those of Isolated HSA and SPIONs as Determined by Fitting of SANS Data

	SPIONs/HSA	SPIONs^a^	HSA
model	core–shell sphere + power law	core–shell sphere	ellipsoid
core radius *r*_core_(Å)	25 ± 2	27 ± 2	
sld core ρ_core_ × 10^6^ (Å^–2^)	6.9	6.9	
shell thickness *d* (Å)	48 ± 1	36 ± 1	
sld shell ρ_shell_ × 10^6^ (Å^–2^)	2.1 ± 0.5	0.62	
sld ρ × 10^6^ (Å^–2^)			1.86
minor radius *a* (Å)			22 ± 0.5
major radius *b* (Å)			72 ± 2.5
power law	–1.5		

a As reported in Luchini et al. *Phys. Chem., Chem. Phys.***2016** , *18*, 18441.

The overall radius of SPIONs/HSA cannot be immediately interpreted as the sum of SPION and HSA radii. The last apparent discrepancy may be due to (i) HSA penetration within NP coating, (ii) removal of the external 18LPC layer, or (iii) the result of an incomplete protein coating. As for the first point, such a drastic change in the HSA environment should have resulted in a much more marked change of protein emission wavelength with respect to what was observed in the fluorescence spectra of SPIONs/HSA. A similar consideration could be done in the second case, too: displacement of 18LPC would leave SPIONs exposing the hydrophobic tails of oleic acid/oleylamine that would then be free to interact with HSA. HSA is a lipid binding protein with many sites devoted to lipid recognition, but also in this case the fluorescence spectra of the HSA/SPIONs should present a blue shift at least comparable to that observed for the 18LPC/HSA system (Figure S3). Thus, considering also the *R*_H_ value determined by DLS, which differs from the radius obtained by SANS for taking into account also the hydration shell, we think that in the SPIONs/HSA system protein molecules are bound on the NP coating forming an incomplete protein corona. The quite high polydispersity of SPION/HSA radii may be a further index of incomplete coating.

It is interesting to note that formation of an incomplete protein shell may be the driving force for NP/protein aggregation, which was proved to happen by both DLS and SANS results. In this respect, this likely represents a different aggregation mechanism with respect to the SPIONs/HTF system where the depletion effect seems preponderant.

**Figure 10:**
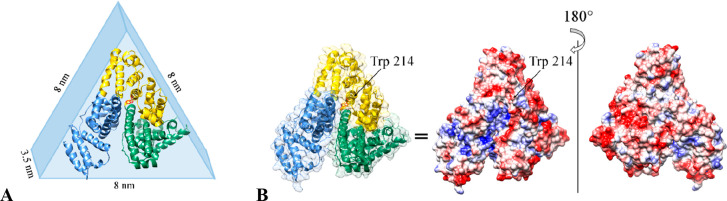
(A) Crystal structure and scheme of HSA with different domains highlighted in different colors. The only Trp is marked in red. (B) HSA electrostatic surface representation at pH 7: positive charges are colored in blue, negative ones in red, and neutral zone in light gray. The position of Trp is explicitly shown.

Based on fluorescence, DLS, and SANS results it is possible to propose a HSA binding mode. HSA has an almost triangular shape, with 8 nm edges and 3.5 nm thickness (Figure 10A). The only tryptophan residue (marked in red in Figure 10A) is located in the middle of one of the HSA faces and is quite exposed to the solvent. The NP quenching effect, as well as the blue shift of the tryptophan emission maximum, suggests a close interaction between the nanoparticle and the tryptophan. At the same time DLS and SANS indicate the presence of a thin protein layer, which is not compatible with HSA binding with any of its 8 nm edges, as observed in the case of some Au/Ag NPs^61^ and Au NPS.^63^ Thus, we can infer that HSA binds SPIONs in a “side-on” mode,^29^ similarly to what found in the case of polymer-coated FePt NPs.^64^ At the same time we can rule out the formation of protein dimers on the NP surface, as suggested for BSA on Al_2_O_3_ NPs.^65^

This hypothesis well justifies the zeta potential results too. Indeed, HSA is characterized by the presence of differently charged patches on its surface. On the Trp face, numerous positive patches may drive the binding via Coulomb interactions^64^ with the slightly negatively charged SPIONs, by leaving the opposite HSA face exposed to the solvent, which is less charged (Figure 10B).

We can tentatively evaluate the number of HSA molecules bound on the NP surface (*N*) considering the dimensions of SPIONs and HSA/SPIONs (Figure S10) as determined by DLS or SANS as
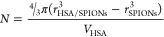
5We find *N* ≈ 7 molecules in both cases, which is in reasonable agreement with results obtained in the case of BSA and different superparamagnetic nanoparticles with similar dimensions.^29^ At the same time, this value is significantly lower than the maximum number of HSA molecules required to form a monolayer on the NP through a side-on binding mode that is about 20 (calculated by dividing the surface area of an individual NP by the triangular area of one HSA molecule^66^), strengthening the hypothesis of incomplete coverage.

### Interaction of SPIONs/HSA Aggregates with Model Membranes

Overall, the characterization of the SPIONs/HSA system points toward the presence of a NP with a slightly different coating, including an incomplete HSA layer strongly adsorbed on the 18LPC. This modification may affect the behavior of NPs *in vivo*, particularly their interaction with cells and internalization pathways. Since the first step of NP entry into cells is determined by their interaction with the cell membrane,^67^ we analyzed the SPIONs/HSA interaction with model membranes by means of neutron reflectivity.^68^ NP membrane activity can be predicted by observing the interactions with solid-supported lipid bilayers, composed of homogeneous fluid lipid mixtures, without raftlike domains or embedded membrane proteins.^67^ Thus, we studied HSA and SPIONs/HSA interaction with lipid bilayers formed by 1-palmitoyl-2-oleoyl-*sn*-glycero-3-phosphocholine (POPC), 1-palmitoyl-2-oleoyl-*sn*-glycero-3-phospho-(1′-rac-glycerol) (POPG), and cholesterol (Chol) at different molar ratios, namely POPC/POPG/Chol 72/8/20 and 56/4/40 mol/mol/mol. POPC is a zwitterionic lipid, while POPG was used to confer a slight negative charge to lipid bilayers, thus better mimicking biological membranes.^69^ Cholesterol was also added as it is an important component of cellular membranes, which regulates their fluidity and packing and takes part in several biological processes. Data were fit according to a box model^53^ with the silicon block, the thin solvent layer interposed between it and the supported bilayer, the inner headgroups, the hydrophobic chains, and the outer headgroups of the bilayer, each of which represents a box. In the case of systems containing either HSA, SPIONs/HSA, or SPIONs, an additional layer was included (Figure S11). Each box was characterized in terms of thickness, *sld*, solvent content, and roughness. Structural parameters of the silicon block were kept fixed and equal to those obtained from the analysis of the bare surfaces.

**Figure 11:**
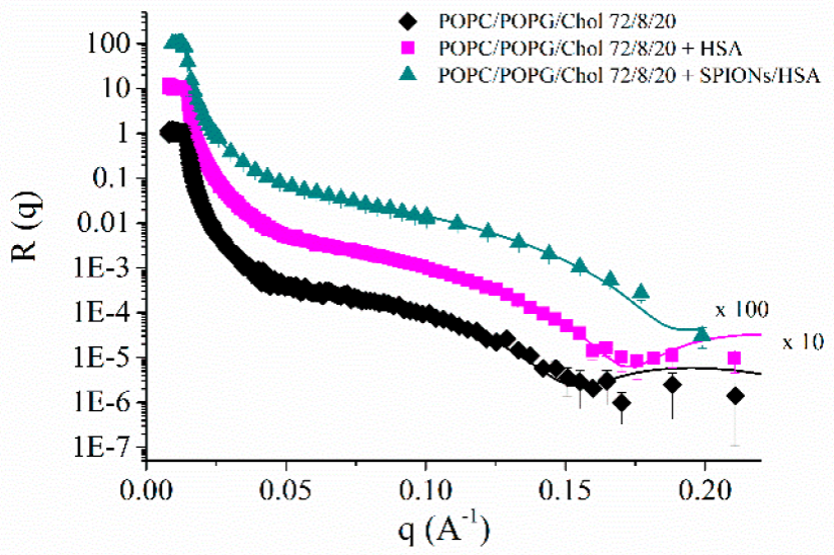
NR profiles for POPC/POPG/Chol 72/8/20 bilayer before and after injection of either SPIONs/HSA or HSA in D_2_O. Best fitting curves are also reported.

As an example, in Figure 11 the NR profiles, both experimental data and fitting curves, of the POPC/POPG/Chol 72/8/20 bilayer alone and in the presence of either HSA or SPIONs/HSA in D_2_O are reported (NR profiles in different contrast media are reported in Figure S12).

Parameters derived from fitting are summarized in Table 2 compared to those reported for the same lipid bilayer in the presence of SPIONs.^69^ These results suggest the adhesion of both SPIONs/HSA and HSA on the surface of the bilayers, even if the high solvent content indicates that only a small fraction is indeed present on the bilayer surface, while most of them are likely removed by washing with solvent.

**Table 2:** Comparison among Structural Parameters of POPC/POPG/Chol 72/8/20 Lipid Bilayer before and after Injection of SPIONs/HSA, HSA Alone, and SPIONs Alone^a^

	thickness (Å)	sld × 10^6^ (Å^–2^)	solvent fraction	roughness (Å)
POPC/POPG/Chol72/8/20				
inner headgroups	7 ± 1	1.65 ± 0.02	0.45 ± 0.01	2 ± 1
acyl chains	34 ± 1	–0.15 ± 0.02	0.010 ± 0.001	6 ± 1
outer headgroups	7 ± 1	1.65 ± 0.02	0.75 ± 0.05	2 ± 1
POPC/POPG/Chol72/8/20 + SPIONs/HSA				
inner headgroups	6.7 ± 0.3	1.7 ± 0.1	0.27 ± 0.02	2 ± 1
acyl chains	32 ± 1	–0.15 ± 0.02	0.025 ± 0.002	3 ± 1
outer headgroups	10 ± 1	1.50 ± 0.04	0.62 ± 0.04	2 ± 1
SPIONs/HSA	80 ± 2	2.65 ± 0.06	0.86 ± 0.02	40 ± 5
POPC/POPG/Chol72/8/20 + HSA				
inner headgroups	6 ± 1	1.60 ± 0.02	0.40 ± 0.05	4 ± 2
acyl chains	31 ± 1	–0.15 ± 0.02	0.21 ± 0.01	3 ± 1
outer headgroups	9 ± 1	1.40 ± 0.02	0.45 ± 0.01	4 ± 1
HSA	15 ± 4	5.00 ± 0.02	0.90 ± 0.02	5 ± 2
POPC/POPG/Chol72/8/20 + SPIONs^b^				
inner headgroups	7 ± 1^b^	1.60 ± 0.02^b^	0.32 ± 0.02^b^	5 ± 1^b^
acyl chains	33 ± 1^b^	–0.15 ± 0.02^b^	0.09 ± 0.01^b^	2 ± 1^b^
outer headgroups	7 ± 1^b^	1.44 ± 0.02^b^	0.45 ± 0.03^b^	7 ± 2^b^
SPIONs	65 ± 5^b^	1.1 ± 0.2^b^	0.90 ± 0.01^b^	25 ± 5^b^

a Errors as derived from fitting are reported.

b As reported in Luchini et al.^69^

In further detail, SPIONs/HSA binding does not affect bilayer features, with a total bilayer thickness of about 49 Å, very similar to the 48 Å of the isolated bilayer. The interaction with HSA does not induce any significant change in the bilayer thickness (46 Å) but, on the other hand, affects the solvent content, which increases with respect to the pure bilayer, particularly in the acyl chain region. This finding may suggest that isolated proteins could slightly enhance membrane permeability. In this respect, it should be recalled that the model membranes used have a slight negative charge, and HSA presents positive residues on its surface; thus, an electrostatic interaction between them may occur. On the other hand, these positive protein patches are shielded in the SPIONs/HSA assemblies (see above), and this may explain the different behavior of the two systems.

Results obtained in the case of POPC/POPG/Chol 56/4/40 generally agree with those illustrated for the POPC/POPG/Chol 72/8/20 system (see Supporting Information Table S1 and Figures S13 and S14).

Overall, NR analysis clearly shows that interaction with SPIONs/HSA does not significantly alter the membrane properties: specifically, they do not break the bilayer or induce the formation of pores. Similar results obtained with SPIONs^69^ pointed toward their biocompatibility, and the present data reinforce this idea since it should be recalled that NPs rarely, if ever, retain their properties when placed in a biological context and most likely what meet membranes are NPs coated by serum proteins, such as HSA.

## Conclusions

Here we show that SPIONs covered by a double layer of oleic acid/oleylamine and 18LPC are able to interact with both the abundant human plasma proteins HSA and HTF, but the kind and strength of interaction is crucially dependent on the nature of the protein.

In the case of HTF the interaction is rather weak. Fluorescence quenching experiments indicate in the HTF/SPIONs system the coexistence of static and dynamic quenching, DLS analysis confirms the interaction between SPIONs and HTF resulting in some sort of NP–protein aggregates, whose dimensions range from *R*_H_ ≈ 20 to 40 nm, but the formation of a stable complex is ruled out by the analysis of washed samples where no CD signal due to the protein is detectable. Notably no effect of SPIONs on HTF secondary and tertiary structure is observed, differently than what is found for bare and PVA-coated SPIONs with similar dimensions, which causes irreversible changes of the protein conformation, even if it should be noted that in that case holo-transferrin was used.^70^

On the other hand, in the case of HSA a tight NP–protein complex forms, characterized by a binding constant of ∼10^8^ M^–1^ and a well-defined geometry of interaction; i.e., HSA binds in a side-on mode. It is interesting that HSA seems able to form both a hard and a soft corona on the SPION surface: indeed in the presence of protein excess we observe a population of *R*_H_ ≈ 25 nm in DLS profiles coexisting with the isolated HSA population. We can suggest that multiple protein layers can form, but when a threshold is reached no further HSA binding occurs and protein molecules remain unbound in solution. Upon extensive washing of the protein excess, only SPION/HSA complexes with *R*_H_ ≈ 10 nm are observed, an indication that loosely bound protein molecules are removed by the washing procedure together with unbound ones.

The presence of hydrophobic moieties as well as hydrophilic negatively charged groups is the basic structural requirement for ligand binding to HSA.^71^ The peculiar coating of our SPIONs, bearing zwitterionic amphiphiles that at physiological pH are characterized by a slightly negative charge, as determined by electrophoretic light scattering, may justify the strong binding of HSA. Furthermore, the driving force for the interaction may be envisaged also in the slight change of the HSA structure in the presence of NPs, as highlighted by the CD spectra. In the case of binding of the analogous protein BSA to negatively charged silica particles, the slight change of protein structure, with an increase of unordered content, was suggested to be the driving force for the binding.^72^ Indeed, this kind of structural reorganization may take place driven by favorable protein–surface interactions and involves an entropy gain due to the loss of ordered secondary structure inside the protein plus the release of counterions or solvation molecules.^73^

Usually strong protein binding to flat surfaces of large NPs was suggested as a cause of protein structure modification and even unfolding.^74,75^ Unfolded or misfolded proteins not only are devoid of their normal biological activity but often aggregate and/or interact improperly with other cellular components, with the result of impairing cell viability and eventually even causing cell death.^74^ In this respect the small dimension of our SPIONs can be called into play for their biocompatibility, at least with respect to interactions with proteins: indeed in the presence of a strong binding between a quite large protein such as HSA and the NP surface, no severe conformational change is detected for the protein.

HSA-covered SPIONs are able to interact with and adhere to the surface of lipid bilayers used as membrane models without removing lipids or affecting the membrane structure, similarly to the reference SPIONs.^69^ Considering the potential application of SPIONs as MRI contrast agents, they should be able to interact with the cells of a target tissue without compromising their life, and this interaction should occur even in the presence of the protein corona very likely formed *in vivo*.

Finally, the apparent preferential binding of HSA with respect to HTF, taking into account the higher HSA concentration in human plasma (40 vs 2.8 mg mL^–1^ for HSA and HTF, respectively),^76^ may have important biological consequences. In fact, a HTF receptor is often overexpressed on the surface of malignant cells;^39^ thus, NP coverage by HTF may significantly affect the fate of the NP *in vivo*. On the other hand, the HSA layer tightly bound to the SPIONs could be exploited for further functionalization of NPs by taking advantage of the carrier properties of this protein, which have often been used to deliver drugs and diagnostic probes.^77−79^ Moreover, SPIONs bearing a protein corona enriched in albumin might better escape the immune system,^80^ and it has been also shown that precoating SPIONs with albumin and lipoproteins can help them to cross biological barriers, such as the blood–brain barrier.^81^

Altogether, the present findings reinforce the idea of our amphiphiles-coated SPIONs as a biocompatible nanodevice for biomedical applications.

## Supporting Information Available

Details on the evaluation of NP concentration and fitting of HSA SANS data, structural parameters derived by NR analysis, HTF fluorescence spectra and overlapped HSA fluorescence spectra in the presence and in the absence of SPIONs, HSA fluorescence spectra in the presence and in the absence of 18LPC, time evolution melting profile of SPIONs/HSA, HSA CD spectra and melting profiles in the presence and in the absence of 18LPC, fluorescence spectra of HSA and HTF at increasing SPION concentration, DLS profiles of HTF and SPIONs/HTF, time evolution of SPIONs/HTF DLS profile, CD spectra of SPIONs/HSA before and after removal of excess protein, HSA SANS profile, schematic representation of SPION, HSA, and SPION/HSA, schematic representation of the box model, and NR profiles of different systems in different contrast media (PDF)
