# Supplementary material for: Interaction with Human Serum Proteins Reveals Biocompatibility of Phosphocholine-Functionalized SPIONs and Formation of Albumin-Decorated Nanoparticles
Source: Langmuir. 2020 Jun 23;36(30):8777–91. doi: 10.1021/acs.langmuir.0c01083 (PMC8008447; doi:10.1021/acs.langmuir.0c01083)
Supplement: Supplementary file 1 — la0c01083_si_001.pdf [file la0c01083_si_001.pdf]

Supporting information

to

Interaction with human serum proteins reveals biocompatibility of phosphocholine-functionalized SPIONs and formation of albumin-decorated nanoparticles

Irene Russo Krauss,[1,2,*] Alessandra Picariello,[1] Giuseppe Vitiello,[2,3] Augusta De Santis,[1,2] Alexandros Koutsioubas,[4] Judith E. Houston,[5] Giovanna Fragneto,[6] Luigi Paduano[1,2,*]

[1]Department of Chemical Sciences, University of Naples Federico II, Naples, Italy

[2] CSGI, Center for Colloid and Surface Science, Sesto Fiorentino (FI), Italy.

[3] Department of Chemical, Materials and Production Engineering, University of Naples Federico II, Naples, Italy

[4] Jülich Centre for Neutron Science (JCNS) at Heinz Maier-Leibnitz Zentrum (MLZ), Forschungszentrum Jülich GmbH, Lichtenbergstraße 1, 85747 Garching, Germany.

[5] European Spallation Source ERIC, Box 176, SE-22 100 Lund, Sweden

[6] Institut Laue-Langevin (ILL), 71 avenue des Martyrs, BP 156, 38042, Grenoble, France

*To whom correspondence should be addressed.

Irene Russo Krauss, Tel: +39 081674227; Email: irene.russokrauss@unina.it,

Luigi Paduano, Tel: +39 081674227; Email: luigi.paduano@unina.it

Number of pages: 19
Number of figures: 14
Number of schemes: 0
Number of tables: 1

Table of Contents

Evaluation of NP concentration.......................................3

Small Angle Neutron Scattering analysis of HSA.......................................3

Table S1 Comparison among structural parameters of POPC/POPG/Chol 56/4/40 lipid bilayer before and after injection of either SPIONs/HSA, HSA or SPIONs. 4

Figure S1 Fluorescence spectra of HTF in the absence and in the presence of SPIONs 5

Figure S2 Overlapped fluorescence spectra of HSA in the absence and in the presence of SPIONs. 6

Figure S3 Comparison between fluorescence spectra of HSA in the absence and in the presence of 18LPC 7

Figure S4 Time evolution of melting profile for SPIONs/HSA 8
Figure S5 Comparison between CD spectra and melting profiles of HSA alone and incubated with 18LPC 9
Figure S6 Fluorescence spectra of HSA and HTF at increasing SPION concentration 10
Figure S7 DLS profiles and time evolution of DLS profiles of SPIONs/HTF 11
Figure S8 Comparison between CD spectra of SPIONs/HSA before and after removal of excess protein 12
Figure S9 SANS profile of HSA. 13
Figure S10 Scheme showing dimensions of SPIONs, HSA and SPION/HSA 14
Figure S11 A scheme of the box-model used for fitting of NR data 15
Figure S12 NR profiles for POPC/POPG/Chol 72/8/20 lipid bilayer upon injection of either SPIONs/HSA or HSA in different contrast media 16
Figure S13 NR profiles for POPC/POPG/Chol 56/4/40 bilayer before and after injection of either SPIONs/HSA or HSA 17
Figure S14 NR profiles for POPC/POPG/Chol 56/4/40 lipid bilayer upon injection of either SPIONs/HSA or HSA in different contrast media 18
References 19

Evaluation of NP concentration

Fe concentration in nanoparticle samples was determined by ICP mass spectrometry in terms of mg mL^{-1} concentration. Considering a Fe_3O_4 density of 5.2 g/cm^3 and the volume of NP core as experimentally determined by SANS it was possible to estimate the weight of a single nanoparticle being 10^{-18} g, corresponding to a nanoparticle molecular weight of about 602200 g/mol. We thus converted Fe concentration into NP molar concentration and used this value to determine the 18LPC concentration needed for functionalization (a procedure employed in previous papers by Luchini et al.) and later on the protein concentration necessary to obtain SPIONs/protein samples. We are aware of the several approximation made, but we referred to an order of magnitude rather than a defined concentration value.

According to this procedure a rough 10-fold difference in mg mL^{-1} concentration of SPIONs and HSA exists in samples with 1:100 NP:protein molar ratio, i.e. when protein concentration is 0.2 mg mL^{-1} as in CD and fluorescence measurements, NP concentration is about 0.02 mg mL^{-1}, when NP concentration is 1 mg mL^{-1} as in DLS measurement HSA concentration is about 10 mg mL^{-1}.

Small Angle Neutron Scattering analysis of HSA

In order to extract structural parameters for HSA, the SANS data have been modeled considering that a collection of charged prolate ellipsoids were present in the system. As form factor, *P(q)*, an ellipsoid of revolution with uniform scattering length density was considered.[1]

The interparticle structure factor of the equivalent sphere *S(q)* used was that calculated by solving the Ornstein-Zernike equation using the closure relation given by the rescaled mean spherical approximation (RMSA).[2-4]

The sld imposed in the fitting was 1.86 10^{-6} $Å^{-2}$. In general, due to hydrogen-deuterium exchange this value can range between 1.86 10^{-6} to 3.14 10^{-6} $Å^{-2}$. However, we choose to consider the protein in its fully hydrogenated form.[5] Parameters obtained by fitting indicate a major radius of 72±2.5 Å, a minor radius of 22±0.5 and an overall charge of -6±1.

The fitting was performed by SasView Version 5.0.2 (Zenodo, 10.5281/zenodo.3752443).

Table S1 Comparison among structural parameters of POPC/POPG/Chol 56/4/40 lipid bilayer before and after injection of either SPIONs/HSA, HSA or SPIONs. Errors as deriving from fitting are reported

* as reported in Luchini *et al*.[6]

	**Thickness (Å)**	***sld*** **x** $\mathbf{10^6}$ **(Å^{-2})**	**Solvent fraction**	**Roughness (Å)**
**POPC/POPG/Chol 56/4/40**				
Headgroups	7±1	1.65±0.02	0.415±0.01	5±1
Acyl chains	34±2	-0.11±0.02	0.35±0.01	7±1
**POPC/POPG/Chol 56/4/40 + SPIONs/HSA**				
Inner Headgroups	6.6±0.2	1.60±0.02	0.15±0.01	2±1
Acyl chains	34±1	-0.11±0.02	0.020±0.003	4±1
Outer Headgroupes	9±1	1.50±0.04	0.64±0.04	2±1
SPIONs/HSA	80±3	2.63±0.05	0.88±0.02	40±5
**POPC/POPG/Chol 56/4/40 + HSA**				
Inner Headgroups	8±2	1.60±0.02	0.38±0.06	3±1
Acyl chains	31±4	-0.11±0.02	0.18±0.01	2±1
Outer Headgroupes	9±1	1.39±0.02	0.44±0.08	4±1
HSA	16±6	5.13±0.2	0.90±0.03	5±1
**POPC/POPG/Chol 56/4/40 + SPIONs***				
Inner Headgroups	7±1*	1.60±0.02*	0.35±0.03*	5±1*
Acyl chains	35±2*	-0.11±0.01*	0.12±0.02*	2±1*
Outer Headgroupes	7±1*	1.40±0.02*	0.55±0.04*	10±2*
SPIONs	70±5*	1.1±0.2*	0.85±0.02*	25±5*

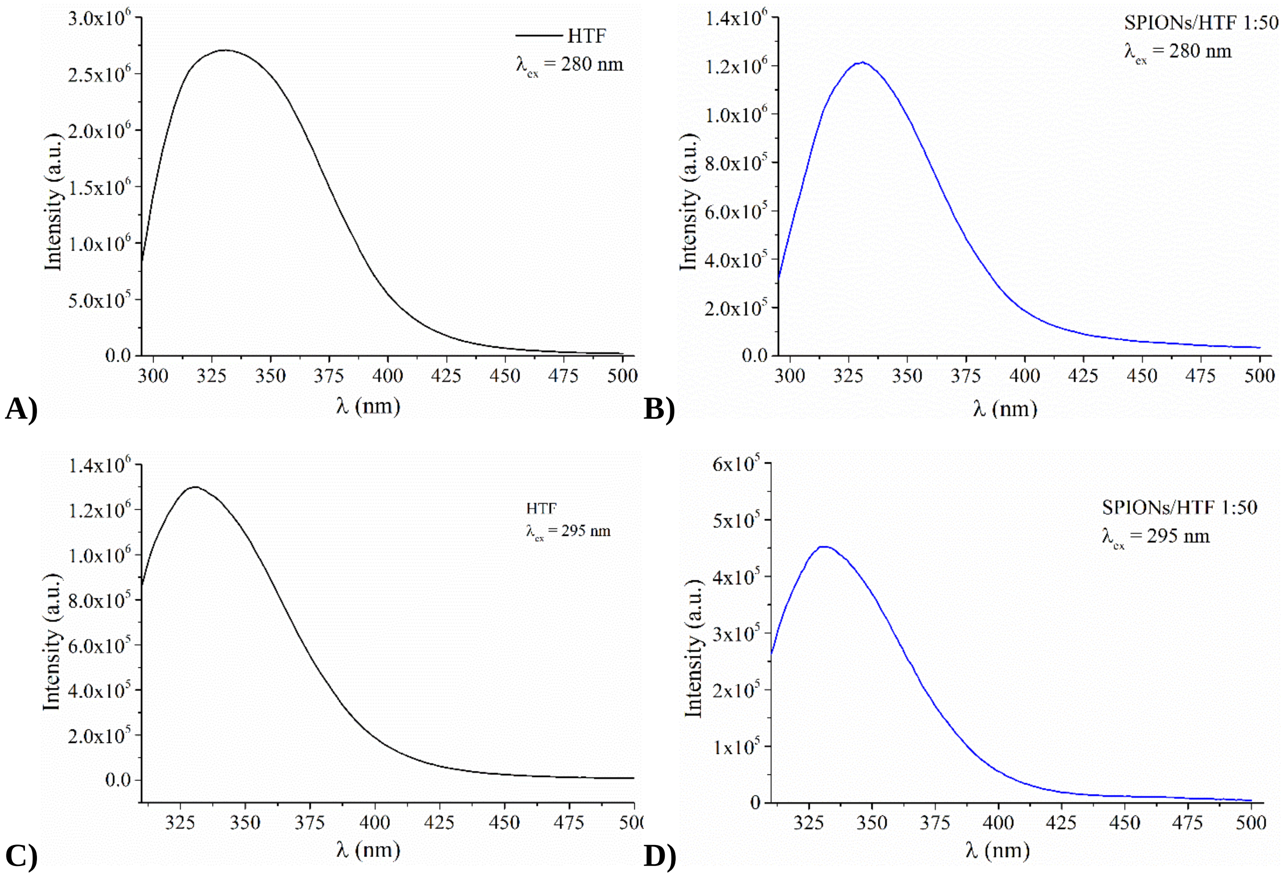

Figure S1 Fluorescence spectra of HTF in the absence (panel A and C) and in the presence of SPIONs at NP:protein molar ratio 1:100 (panel B and D) obtained by exciting at either λ_{exc} = 280 nm (panels A and B) or λ_{exc} = 295 nm (panels C and D).

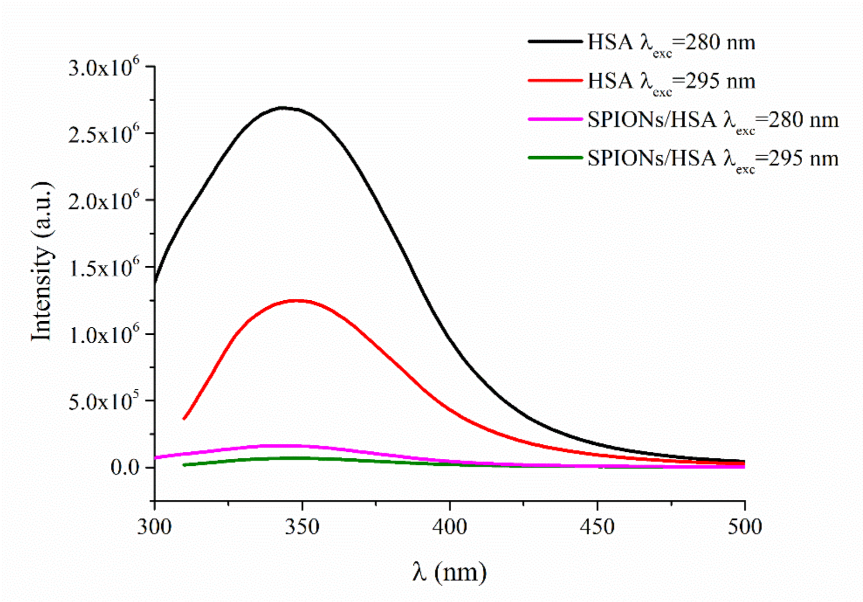

Figure S2 Overlapped fluorescence spectra of HSA in the absence and in the presence of SPIONs at 1:50 NP:protein ratio obtained by exciting at either λ_{exc} = 280 nm or λ_{exc} = 295 nm

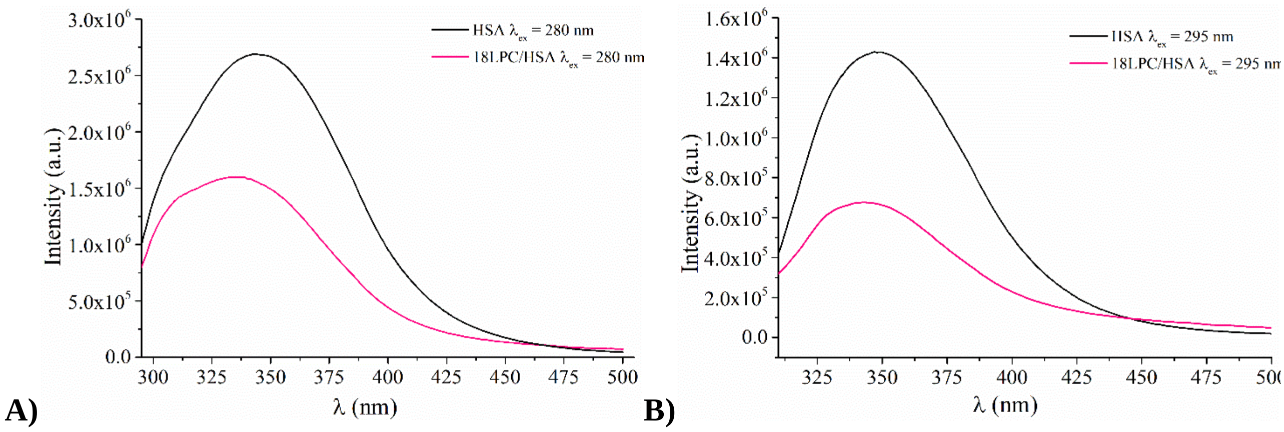

Figure S3 Comparison between fluorescence spectra of HSA in the absence and in the presence of 18LPC obtained by exciting at λ_{exc} = 280 nm (panel A) or 295 nm (panel B).

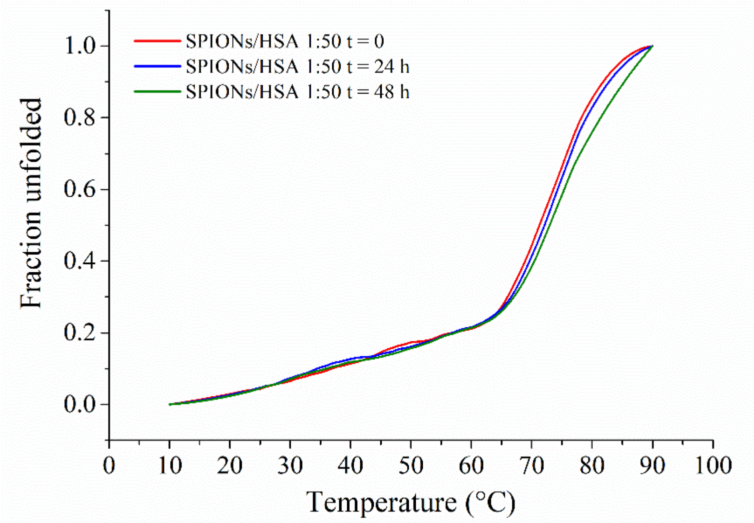

Figure S4 Time evolution of melting profile as obtained by analysis of CD signal at 222 nm for SPIONs/HSA at NP:protein 1:50 molar ratio

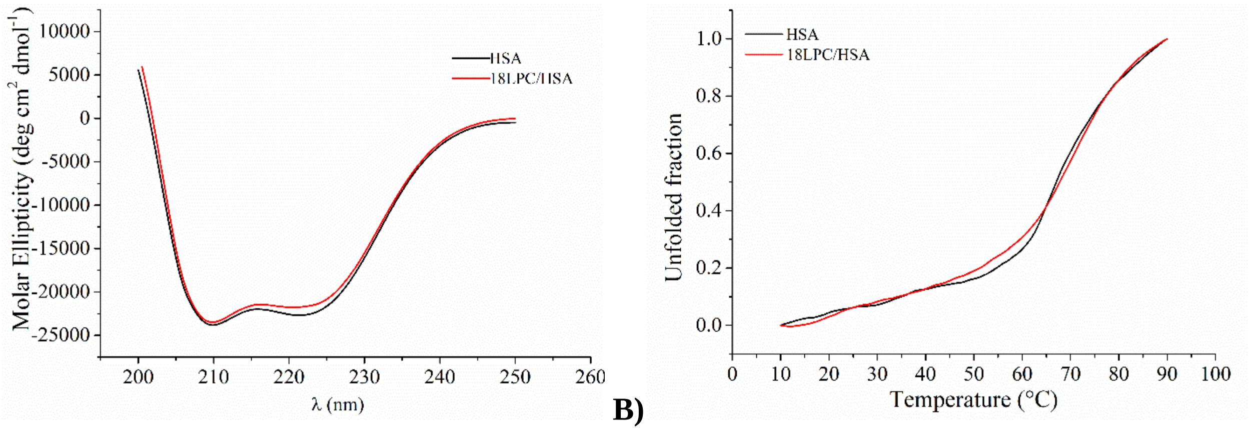

A) B)

Figure S5 Comparison between CD spectra (panel A) and melting profiles as obtained by analysis of CD signal at 222 nm (panel B) of HSA alone and incubated with 18LPC

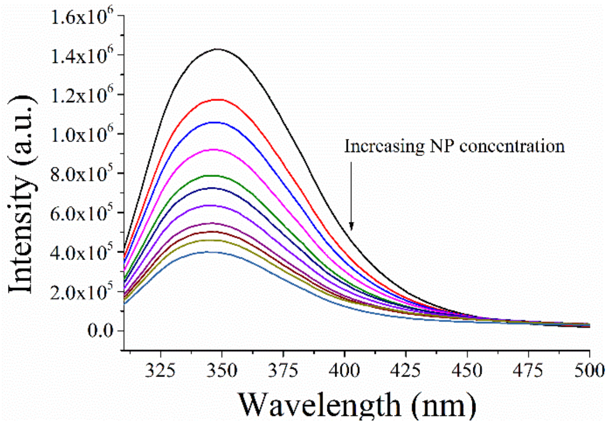

A)

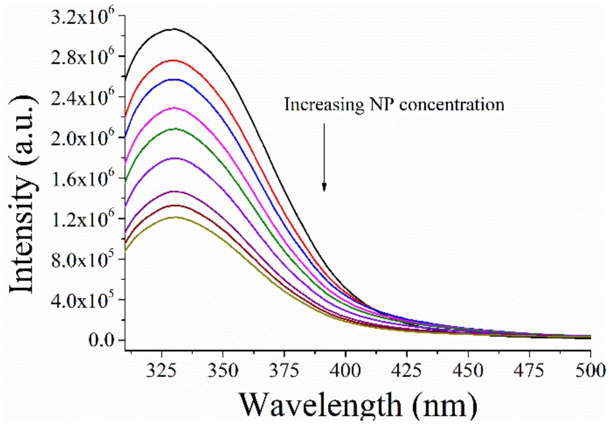

B)

Figure S6 Fluorescence spectra of HSA (panel A) and HTF (panel B) at increasing SPION concentration obtained by exciting at λ_{exc} = 295 nm.

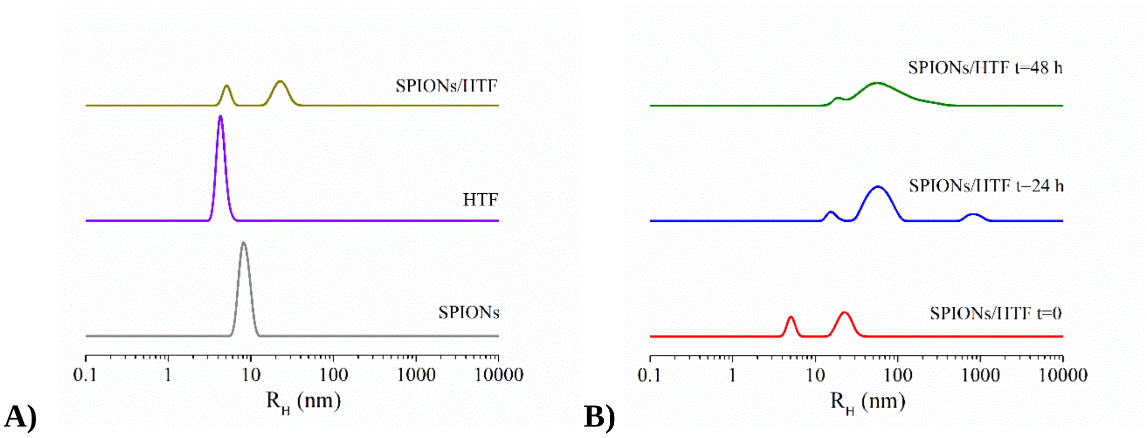

Figure S7 DLS profiles of SPIONs/HTF with respect to isolated SPIONs and protein (panel A); time evolution of DLS profiles of SPIONs/HTF during two days observation (panel B).

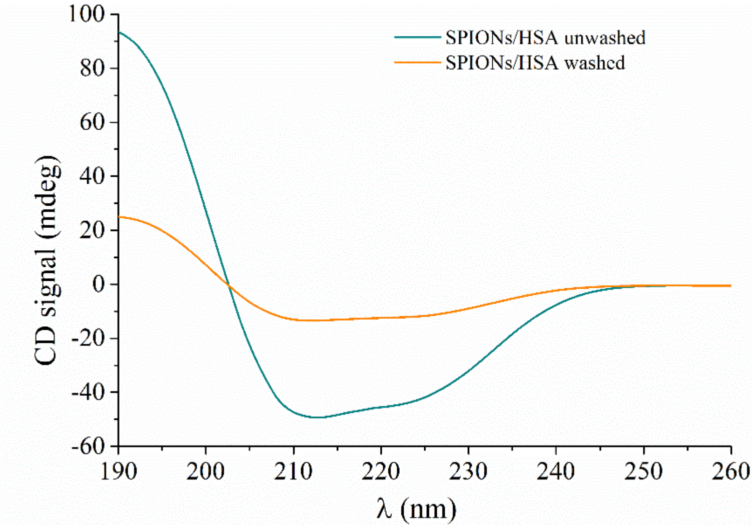

Figure S8 Comparison between CD spectra of SPIONs/HSA before and after removal of excess protein.

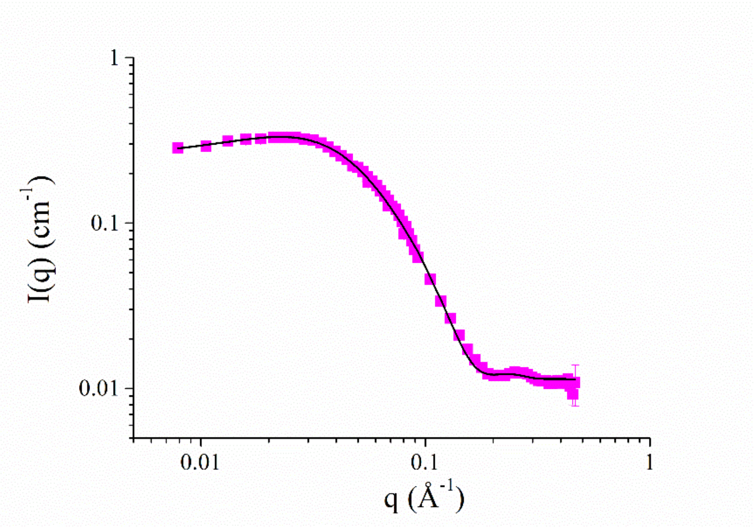

Figure S9 SANS profile (experimental data ■ and best fitting curve (solid line)) of HSA.

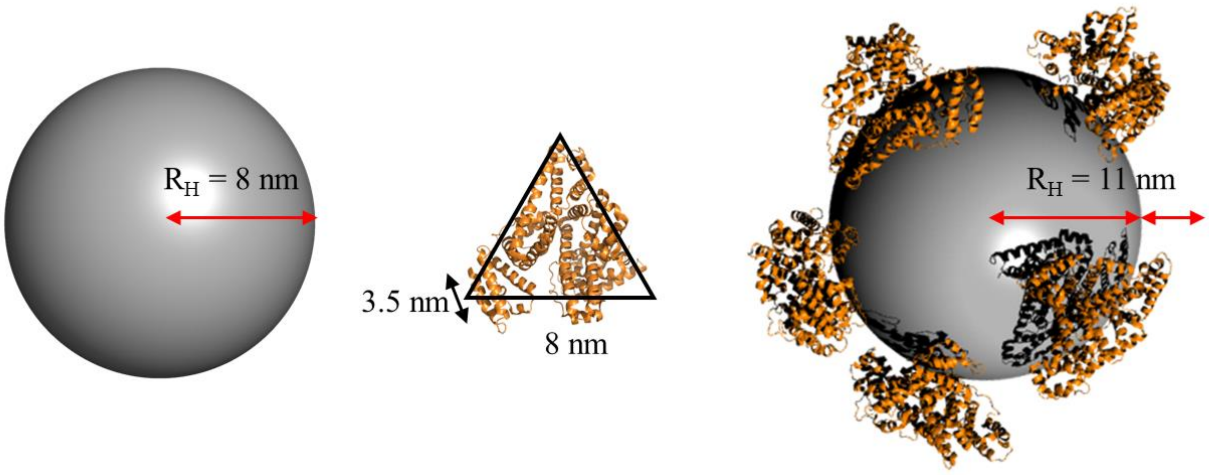

Figure S10 Scheme showing dimensions of SPIONs, HSA as an equilateral prism and SPION/HSA

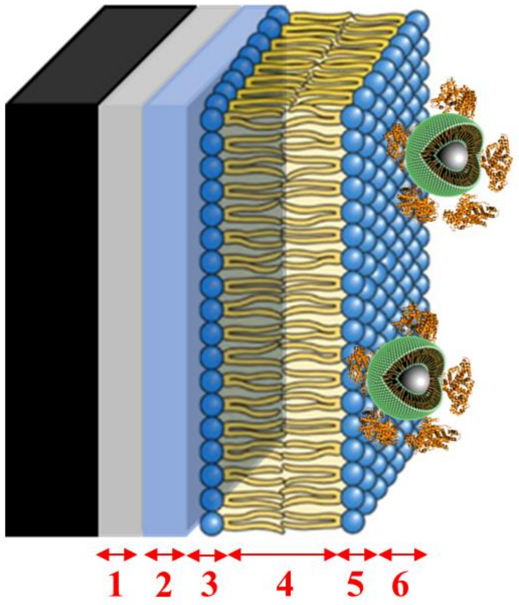

Figure S11 A scheme of the box-model used for fitting of NR data. The silicon block (1), the thin solvent layer interposed between it and the supported bilayer (2), the inner headgroups (3), the hydrophobic chains (4) and the outer headgroups of the bilayer (5) represent each a box. In the case of systems containing either HSA, SPIONs/HSA or SPIONs an additional layer (6) was included

A) 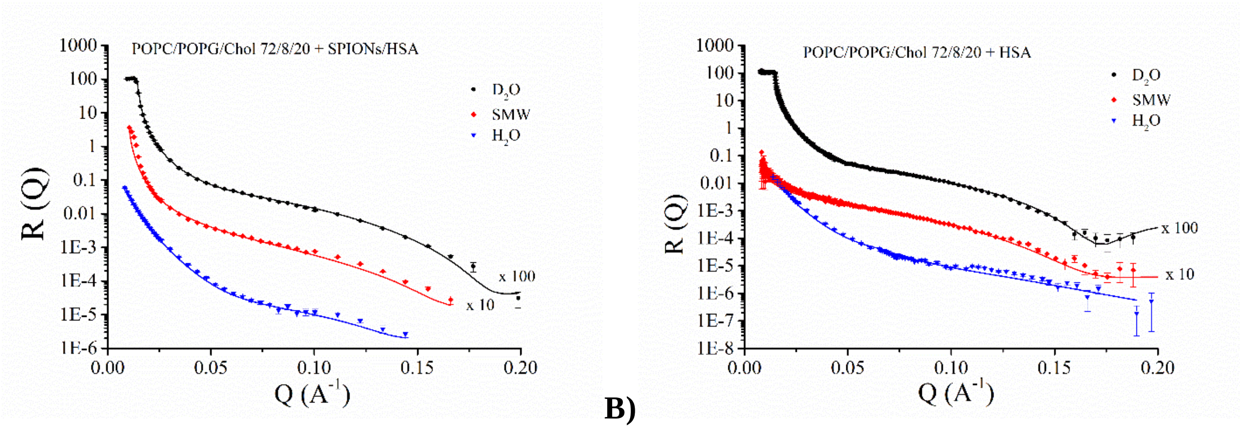

Figure S12 NR profiles for POPC/POPG/Chol 72/8/20 lipid bilayer upon injection of either SPIONs/HSA (panel A) or HSA (panel B) in three different contrast media, i.e. D_2O, H_2O SMW.

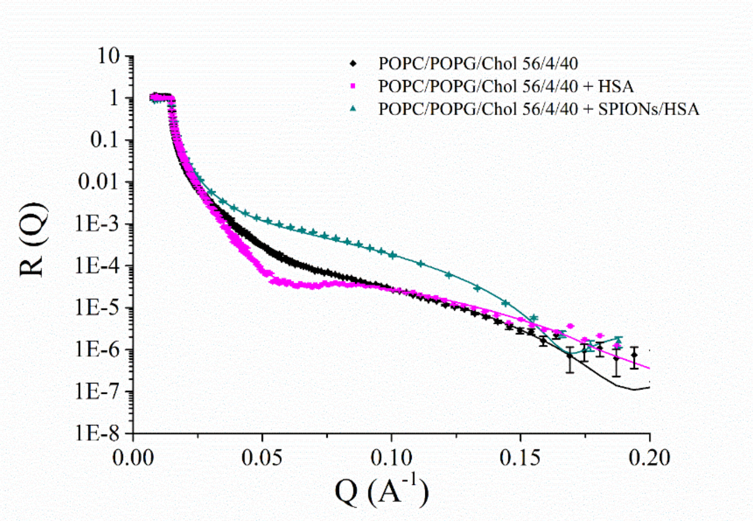

Figure S13 NR profiles for POPC/POPG/Chol 56/4/40 bilayer before and after injection of either SPIONs/HSA or HSA in D_2O. Best fitting curves are also reported.

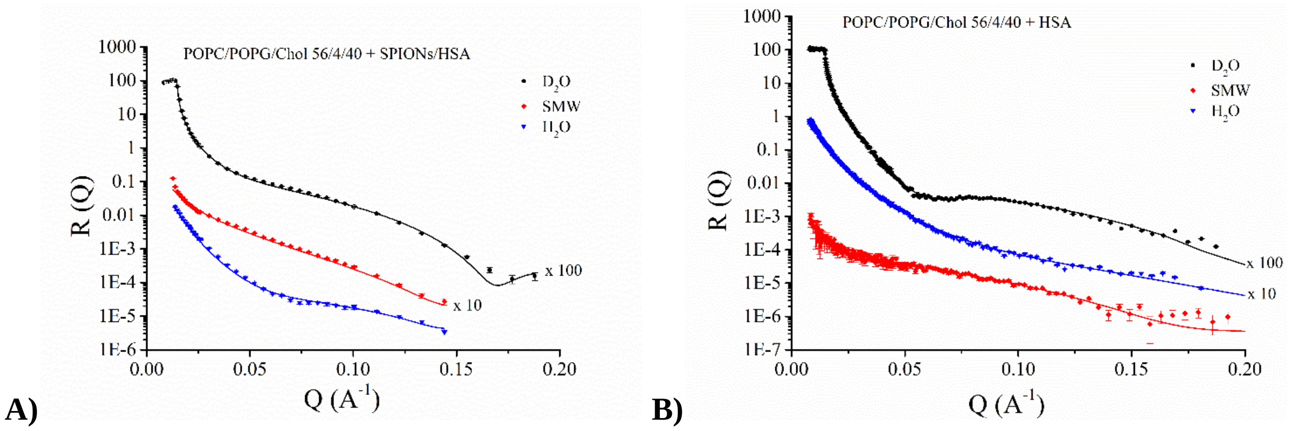

Figure S14 NR profiles for POPC/POPG/Chol 56/4/40 lipid bilayer upon injection of either SPIONs/HSA (panel A) or HSA (panel B) in three different contrast media, i.e. D_2O, H_2O SMW.

References

1. Feigin, L. A.; Svergun, D. I., *Structure Analysis by Small-Angle X-Ray and Neutron Scattering*. Plenum Press: New York, 1987.
2. Hansen, J. B.; Hayter, J. B., A rescaled MSA structure factor for dilute charged colloidal dispersions. *Mol Phys* **1982,** *46*, 651-656.
3. Hayter, J. B.; Penfold, J., An analytic structure factor for macroion solutions. *Mol Phys* **1981,** *42*, 109-118.
4. Hayter, J. B.; Penfold, J., Self-consistent structural and dynamic study of concentrated micelle solutions. *J Chem Soc, Faraday Trans* **1981,** *77*, 1851-1863.
5. Berts, I.; Fragneto, G.; Porcar, L.; Hellsing, M. S.; Rennie, A. R., Controlling adsorption of albumin with hyaluronan on silica surfaces and sulfonated latex particles. *J Colloid Interface Sci* **2017,** *504*, 315-324.
6. Luchini, A.; Gerelli, Y.; Fragneto, G.; Nylander, T.; Palsson, G. K.; Appavou, M. S.; Paduano, L., Neutron Reflectometry reveals the interaction between functionalized SPIONs and the surface of lipid bilayers. *Colloids Surf B* **2017,** *151*, 76-87.
